# Prevalence of Breastfeeding in Infants With Down Syndrome: A Systematic Review and Meta‐Analysis

**DOI:** 10.1111/apa.70628

**Published:** 2026-07-03

**Authors:** Mélina Balland, Julia Prillard, Irène Loras‐Duclaux, Caroline Giroudon, Amélie Zelmar, Damien Sanlaville, Mikail Nourredine

**Affiliations:** ^1^ Hospices Civils de Lyon Lyon France; ^2^ Hospices Civils de Lyon Hôpital Femme Mère Enfant, Service d’hépato‐gastro‐entérologie et nutrition pédiatrique Bron France; ^3^ Hospices Civils de Lyon Service de la Documentation Centrale, TAC Lyon France; ^4^ Service Recherche et Epidémiologie Cliniques, Pôle de Santé Publique Hospices Civils de Lyon Lyon France; ^5^ Hospices Civils de Lyon Service de Génétique Lyon France; ^6^ Université Claude Bernard Lyon 1 Physiopathologie et Génétique du Neurone et du Muscle, UMR5261, CNRS U1315, INSERM Institut NeuroMyoGène Lyon France; ^7^ Hospices Civils de Lyon Service de Biostatistique‐Bioinformatique Lyon France; ^8^ Université Claude Bernard Lyon 1 LBBE, UMR 5558, CNRS, Vas Villeurbanne France

**Keywords:** breastfeeding, Down syndrome, feeding behaviour, meta‐analysis, systematic review

## Abstract

**Aim:**

To estimate the prevalence of breastfeeding—overall, exclusive, partial and depending on infants' age—in infants with Down syndrome, and to investigate associated factors.

**Methods:**

A systematic literature search was conducted in Medline, Cochrane Library, Web of Science, Embase, CINAHL and SciELO up to 1 August 2024. Original articles that estimated the prevalence of breastfeeding in infants with Down syndrome, written in French, English or Spanish, were included. Study quality was assessed using the Joanna Briggs Institute (JBI) scale. Meta‐analyses were performed for breastfeeding outcomes and meta‐regression explored heterogeneity. The review was registered in PROSPERO (CRD42021278019).

**Results:**

Twenty‐six studies (3463 infants) were included. The estimated prevalence of overall breastfeeding regardless of duration was 71.6% (95% CI [60.3; 80.7]; 25 studies, 3351 infants) with high heterogeneity, *I* ^2^ = 94%. The estimated prevalence of exclusive breastfeeding was 38.4% (95% CI [22.4; 57.3]; 10 studies, 1099 infants). No factor assessed in meta‐regression was significantly associated with overall breastfeeding.

**Conclusion:**

The estimated breastfeeding prevalence in infants with Down syndrome is similar to that reported in the general population, despite high heterogeneity. Further studies using standardised methodology to assess breastfeeding barriers and facilitators in the context of Down syndrome would allow us to improve support for breastfeeding.

## Summary

- Breastfeeding is particularly beneficial to infants with Down syndrome, reducing the risk for infections and many related comorbidities such as leukaemia, obesity, or type 1 diabetes.
- Literature suggests that infants with Down syndrome were at risk of breastfeeding difficulty due to hypotonia, weak sucking reflex, and congenital diseases requiring hospitalisation.
- Our meta‐analysis estimated a prevalence of breastfeeding in infants with Down syndrome similar to that of the general population, despite high heterogeneity.

**Abbreviations**
BFHI: Baby‐friendly Hospital Initiative
CI: confidence interval
CINAHL: Cumulative Index to Nursing and Allied Health Literature
JBI: Joanna Briggs Institute
PRISMA: Preferred Reporting Items for Systematic Reviews and Meta‐analyses
SciELO: Scientific Electronic Library Online
UNICEF: United Nations International Children's Emergency Fund
WBTI: World Breastfeeding Trends Initiative
WHO: World Health Organisation

## 1 Introduction

Down syndrome, also known as trisomy 21, is a genetic disorder affecting approximately1/1000 newborns worldwide and caused by the presence of an extra chromosome 21 [1]. Down syndrome is the most common chromosomal condition associated with intellectual disability [2], and patients present with many related comorbidities such as leukaemias [3], obesity [4] and diabetes [5], as well as a greater risk for infections [6, 7, 8]. Early and appropriate care can greatly improve the quality of life in children with Down syndrome [9], involving a close cooperation between the parents and a multidisciplinary medical team. Among beneficial practices, breastfeeding was reported to reduce the risk of the above‐mentioned comorbidities in the general population [10, 11, 12, 13] and to have a positive effect on cognition [14, 15], highlighting its expected benefits for this population.

However, it has been suggested that infants with Down syndrome were at risk of breastfeeding difficulty because of malformations or congenital diseases requiring surgery or hospitalisation, including congenital heart disease, gastrointestinal disorders and intra‐uterine growth restriction [16, 17]. Moreover, a significant proportion of these infants have hypotonia and abnormal oropharyngeal structures, which leads to a weak sucking reflex and subsequent swallowing difficulty [18]. These factors often lead to sucking difficulties, making breastfeeding challenging or unsustainable. Other common barriers to breastfeeding in infants with Down syndrome included the failure to provide feeding on demand, as these infants may feed less frequently, and the use of improper breastfeeding techniques [19]. In addition, maternal‐related barriers were also identified herein such as perceived insufficient milk supply and mental health concerns including frustration, depression and anxiety [16].

The multiple challenges in initiating and maintaining breastfeeding in infants with Down syndrome suggest that this specific population may be less frequently breastfed and during a shorter period compared to the general population [20]. However, to our knowledge, due to the few available studies, the current literature lacks sufficient and precise data on breastfeeding practices in children with Down syndrome. To date, no systematic review including a meta‐analysis on the prevalence of breastfeeding in infants with Down syndrome has been published. While Magenis et al. [21] reviewed the topic, they did not perform a meta‐analysis.

The aim of the present study was therefore to estimate the prevalence of breastfeeding in infants with Down syndrome through a systematic literature review and meta‐analysis. In addition, the prevalence of exclusive and partial breastfeeding as well as the prevalence of breastfeeding depending on the infants' age were estimated, and factors potentially associated with breastfeeding were investigated.

## 2 Methods

### 2.1 Protocol and Registration

The protocol of this review was pre‐registered in PROSPERO in February 2024 (CRD42024509743). Amendments to the pre‐registered protocol and post hoc analyses are described below. Preferred Reporting Items for Systematic Reviews and Meta‐analyses (PRISMA) recommendations were followed, as presented in the Table S6, since the PRISMA extension for prevalence meta‐analysis is still under development [22].

### 2.2 Eligibility Criteria

Eligible studies were those including either infants with Down syndrome or mothers of infants with Down syndrome (pre‐natal or post‐natal diagnostic), with or without comorbidity, that reported the number of breastfed infants or extractable breastfeeding percentage. Breastfeeding could be self‐reported by the parents or reported by a healthcare professional or found in a medical record. Interviews of children with Down syndrome were excluded. Data collection could be retrospective, without limit of age of infants with Down syndrome at data collection. Original studies comprising cohort, case control, or cross‐sectional studies published in a peer‐reviewed journal were included. Studies including mixed samples of infants with and without Down syndrome without subgroup analysis for infants with Down syndrome as well as studies involving mothers of children with chromosomal disorders other than Down syndrome were excluded. Case reports, narrative reviews, letters, guidelines and randomised controlled trials were excluded. We only included studies written in English, French and Spanish without restriction regarding the date of publication.

### 2.3 Information Sources

The search strategy and syntax were designed by an information specialist (C.G.). We searched MEDLINE (NLM), Cochrane Library (Wiley), Web of Science (Clarivate), Embase (Elsevier), CINAHL (EBSCO Information Services) and SciELO (FAPESP and BIREME) from 13 December 2023 up to 01 August 2024. The full search strategy and syntax are reported in the Table S3. We created automatic alerts on MEDLINE, Cochrane Library, Web of Science and Embase for new studies published during the review.

### 2.4 Study Selection and Data Collection Processes

Two independent reviewers (M.B., J.P.) conducted the literature search. Each reviewer verified the relevance of the different records based on titles and abstracts. Full texts were then read to determine eligibility. Disagreements were solved through discussions or referred to a third author (M.N.). The selection process was performed using Covidence [23]. Data collection was performed independently for each included study by two reviewers (M.B., J.P.) using a standardised extraction form. The extracted variables included study design, sample size, sampling technique, population characteristics, methods used to evaluate breastfeeding and facilitators and barriers of breastfeeding.

### 2.5 Bias Assessment

The quality of the included studies and critical appraisal was assessed independently by two reviewers (M.B., J.P.) using the Joanna Briggs Institute scale (JBI) [24]. The JBI checklist is composed of nine items: sample representativeness, appropriateness of sampling strategy, sample size adequacy, subject and setting description, data analysis coverage, validity of condition identification, reliability of condition measurement, appropriateness of statistical analysis and response rate adequacy. For each item, the two reviewers assessed whether the criterion was present, absent, or marked as ‘unclear’ when insufficient details were provided (Table S4).

### 2.6 Data Items

The primary objective was to estimate the prevalence of overall breastfeeding in infants with Down syndrome, regardless of the type of breastfeeding or the age of infants with Down syndrome. Secondary objectives were: (1) to estimate the prevalence of exclusive and partial breastfeeding regardless of the age of infants with Down syndrome; (2) to estimate the prevalence of exclusive, partial and overall breastfeeding depending on infants' age groups categorised into exclusive intervals as follows: < 1 month, 1 month included to 3 months, 3 months included to 6 months and ≥ 6 months; and (3) to investigate factors potentially associated with overall breastfeeding.

Breastfeeding was defined as an infant who received breastmilk, either directly from the breast or expressed breastmilk [25]. Of note, overall breastfeeding comprises exclusive, mixed or unspecified breastfeeding. Exclusive breastfeeding was defined as an infant who receives only breastmilk, without other food or drink, while partial breastfeeding was defined as an infant who receives breastmilk along with other food or drink (e.g., artificial milk, complementary food), according to the World Health Organisation (WHO) definition [25]. For longitudinal studies, only the proportion from the first time point was extracted.

Regarding factors potentially associated with overall breastfeeding in infants with Down syndrome, the following variables were selected based on the data found in the literature: proportion of premature infants and proportion of hospitalisation during their first year of life, age of the mothers at delivery, proportion of mothers who during pregnancy reported a wish to breastfeed, proportion of mothers who attended tertiary education, proportion of primiparity, percentage of mothers who received promotion and support of breastfeeding from the nursing staff, level of implementation of breastfeeding policies and practices in the country according to the World Breastfeeding Trends Initiative score (WBTI; /100) [26], and the year during which the data was collected (the median when several years of collection; data publication when not specified).

### 2.7 Data Analysis

Data were analysed using R, version 4.3.3, with the ‘meta’ package, version 4.15‐1 (R Foundation for Statistical Computing, Vienna, Austria). Random‐effect models using generalised linear mixed models with a logit transformation were used for the estimation of the prevalence (if at least five studies reported the outcome) [27]. The 95% confidence intervals (CI) were estimated using standard normal quantile. Heterogeneity was assessed by *I* ^2^ and *τ* ^2^ statistics (estimated by maximum likelihood). Regarding identification of factors potentially associated with overall breastfeeding, the meta‐regression was performed when at least five studies contained the variables of interest. In addition, a subgroup analysis was performed to assess a potential bias related to the age of the children during data collection (< 5 years versus ≥ 5 years, arbitrarily chosen).

### 2.8 Protocol Modifications and Post Hoc Analyses

A sensitivity analysis was performed on the primary outcome by excluding three studies: two studies that included specific populations, infants hospitalised in intensive care [28], or infants undergoing surgery for congenital heart defects [29], and one study that focused on the evaluation of breastfeeding techniques and duration [30] (Figure 3). In addition, four studies conducted before 2001 were also excluded, which is the year when the WHO issued its recommendation for exclusive breastfeeding during the first 6 months of life.

A post hoc subgroup analysis was added according to the country and to the geographical region (South America, Europe and other). An additional post hoc meta‐regression was conducted based on the percentage of infants with congenital heart disease.

Due to the lack of data, it was not feasible to estimate the prevalence of exclusive breastfeeding and partial breastfeeding depending on the age of the infants with Down syndrome, and to perform the meta‐regression for the variable ‘proportion of mothers who reported a wish to breastfeed during pregnancy’. As opposed to the PROSPERO protocol, the language of the included studies was restricted to English, French and Spanish, and the mean duration of overall breastfeeding was not assessed due to the lack of data.

## 3 Results

### 3.1 Characteristics of the Included Studies

**FIGURE 1:**
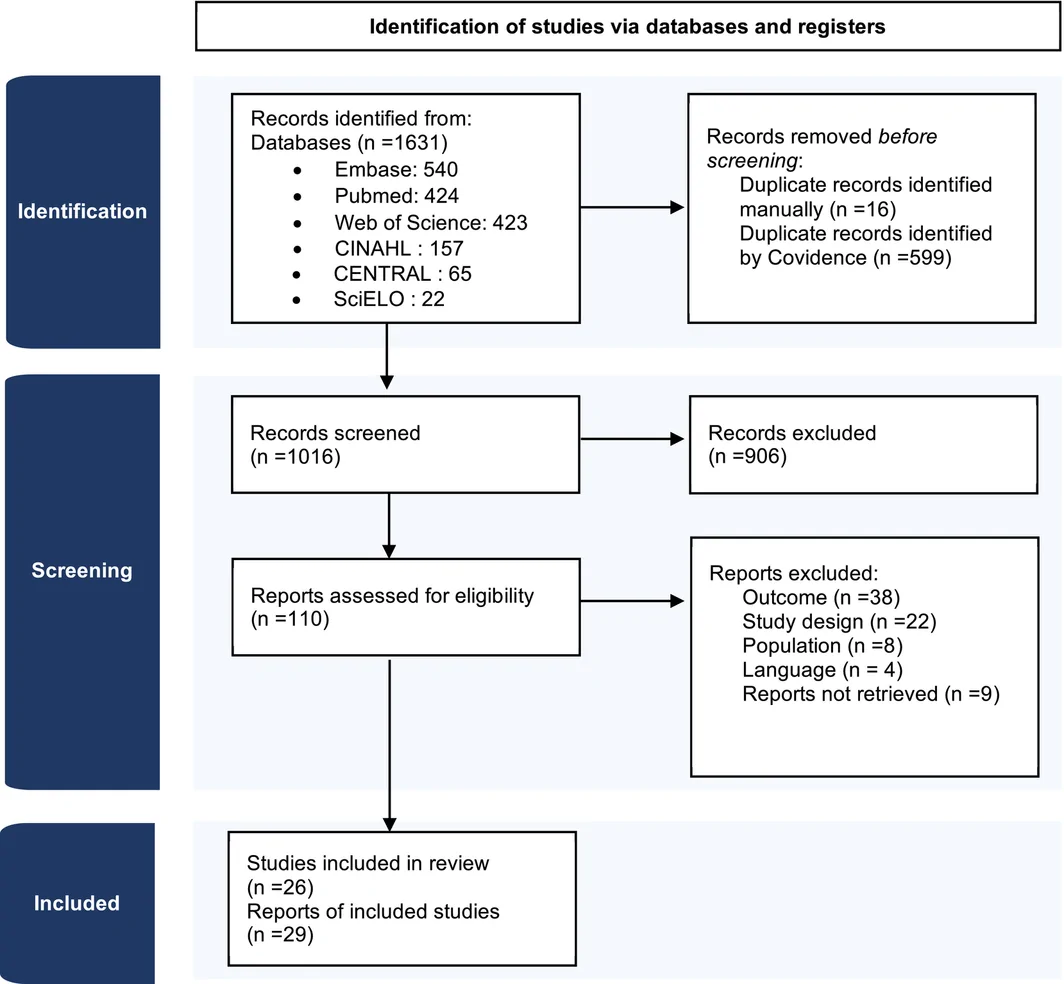
Flow chart.

A total of 1631 records were identified, and after removing duplicates (*n* = 615), 1016 records were selected for screening. Following this screening, 110 reports were eligible based on title and abstract to be read in full‐text. Among the 110 reports that underwent full‐text screening, 81 were excluded (Table S5). Two studies were available only as abstracts, but they provided sufficient data for eligibility criteria and primary outcome. Overall, 29 reports were included in this systematic review, resulting in 26 studies. Among them, 25 studies were included in the meta‐analysis for the primary outcome, as Oliveira et al. had data only for a secondary outcome (Figure 1).

### 3.2 Overall Characteristics of the Studies

Twenty cross‐sectional studies, five longitudinal studies, and one case–control study were included. Characteristics of the included studies are presented in Table 1. The studies were published between 1984 and 2024, and sample sizes ranged from 14 to 560, resulting in a total of 3463 infants with Down syndrome or mothers of infants with Down syndrome. Cohorts from 16 different countries were included, mainly from Europe (46%) and South America (27%). Most individuals were recruited from healthcare facilities. Breastfeeding assessment methods included parental questionnaires (46%), reports from medical or paramedical staff (8%), interviews with parents (23%), and analysis of medical records (19%). In cross‐sectional studies based on retrospectively collected data, the mean age of children at the time of data collection ranged from 3 months to 10 years.

**TABLE 1:** Characteristics of the included studies.

Characteristics of the studies									
First author	Publication year	Year of data collection	Country	Study design	Type of recruitment	Sample size (*)	Method to evaluate BF	Age of children at data collection^†^: mean or median ± SD (range)	Score JBI (/9)
Agostini [31]	2021	2019	Brazil	Cross‐sectional	Children with DS followed in referral cardiology hospital	62	Questionnaire to parents	Median 2.5 years (0–5)	6
Aguilar‐Cordero [30]	2019	2015	Spain	Longitudinal *controlled*	Children with DS hospitalised in the University Clinical Hospital San Cecilio in Granada	40	Observation of BF technique by healthcare professionals	Data collected prospectively	5
Al‐Sarheed [32]	2005	Na	Saudi Arabia	Cross‐sectional	Children with DS schooled in 3 schools for children with mental disabilities	225	Questionnaire to parents	Na	8
Aumonier [33, 34]	1984 and 1983	1976–1978	United Kingdom	Longitudinal	Children with DS born in the 10 area health authorities of Greater Manchester	61 (54)	Interviews to parents by visitor every 6 weeks until 18 months, then every 12 weeks until 2 years	Data collected prospectively	7
Bhatia [35]	2005	Na	India	Cross‐sectional *controlled*	Children with DS followed in All India Institute of Medical Sciences of New Delhi	40	Interviews with semi‐structured questionnaire to parents by healthcare professional	Mean: 3 years	6
Bialek‐Dratwa [36]	2023	2020–2021	Poland	Cross‐sectional	Parents of children with DS received an online questionnaire in forums and support groups	195	Questionnaire to parents	Mean: 6.6 years +/− 5.8; median: 4.5 years (0.5–30)	7
Colon [37]	2009	Na	Puerto Rico	Cross‐sectional	Children with DS followed in a specialised institution	26	Interviews with semi‐structured questionnaire to parents by one interviewer	< 12 months	8
Da Cruz Alfaro [28]	2024	2014–2018	Brazil	Retrospective cohort	Children with DS admitted to 3 neonatal intensive care unit in Rio de Janeiro	112	Medical records	Data collected prospectively	5
Enoch [38]	2021	2016–…	United Kingdom	Longitudinal	Children with DS born in the UK since 2016	329	Na	Data collected prospectively	1
Ergaz‐Shaltiel [39]; Padilla [40]	2017	2000–2010	Israel	Retrospective cohort	Children with DS born in the four main medical centres in Jerusalem between 2000 and 2010	403 (301)	Medical records	Data collected prospectively	6
Flores‐Lujano [41]	2009	1998–2006	Mexico	Case–control	Cases: Children with DS followed in six public institutions treating paediatric cancer in Mexico City Controls: Children with DS followed in multiple attention centres or specialised centres for special education	275	Interview with questionnaire for parents by nurse	Median: 82 months (1–227)	7
Génova [42]	2018	2015–2016	Chile	Cross‐sectional	Children with DS followed in the DS Health Care Program of UC Christus Health Network	73	Online questionnaire for parents	(6–24) months	7
Glivetic [43]	2015	2009–2012	Croatia	Retrospective cohort *controlled*	Data from birth and perinatal death certificates for DS cases of all health institutions in Croatia	120	Medical records	Data collected prospectively	5
Hansen [44]	2011	Na	United States of America	Cross‐sectional	Mothers of children with DS received an online questionnaire in websites of 3DS organisations	98	Online questionnaire for mothers	Na	2^‡^
Hopman [45]	1998	Na	Netherland	Cross‐sectional *controlled*	Parents of children with DS member of the Dutch Down Syndrome Foundation's invited to participate in a study on cow's milk protein intolerance	44 (42)	Open interview for parents by nutritionist	Mean: 21 +/− 11 months (max: 4 years)	7
Magenis [21]	2017	Na	Brazil	Cross‐sectional *controlled*	Children with DS attending the association of Parents and Friends of Expectational	19	Interview with questionnaire for parents	Mean: 9.94 years (5–18)	6
Murdoch [46]	1991	Na	New Zealand	Cross‐sectional	Mothers of children with DS born between 1972 and 1988 attended a teleconferenced seminar of the New Zealand Down's Association	117	Questionnaire for parents	(3–19) years	5
Oliveira [47]	2010	Na	Brazil	Cross‐sectional	Children with DS followed in Rio de Janeiro Children's Hospital	112	Interview with questionnaire for mothers by healthcare professional	Mean: 8.3 years; median: 7 years (3–18)	7
Oulmane [48]	2024	2014–2017	Morocco	Cross‐sectional *controlled*	Parents of children with DS attending 11 associations and health centres for children with DS in Marrakech region	279 (278)	Questionnaire for parents	Mean: 10.29 years +/− 6.68	4
Pentony [29]	2023	2019–2020	Ireland	Retrospective cohort *controlled*	Data from medical files of children with DS who underwent surgical repair of cAVSD or VSD in the Children's Health Ireland at Crumlin	60	Medical records	Data collected prospectively	4^‡^
Pisacane [20]	2003	2002	Italy	Cross‐sectional *controlled*	Children with DS followed in university hospitals of Naples, Rome, Catanzaro and Palermo in 2000 and 2001	560	Interviews with the mothers for feeding habits/medical records for perinatal events	Median: 7 years (1–25)	8
Rodriguez Martinez [49]	2010	1999–2008	Spain	Retrospective cohort *controlled*	Children with DS followed in gastro‐enterology at Virgen del Rocio Hospitals in Seville between 1999 and 2008	28	Medical records	Data collected prospectively	5
Rogers [50]	2022	Na	United Kingdom	Cross‐sectional	Mothers of children with DS recruited online or in person through local charities, support groups and social media	40	Questionnaire for parents	Mean: 3.3 months (7–63)	8
Spender [51]	1996	Na	United Kingdom	Cross‐sectional *controlled*	Children with DS registered on the Oxfordshire Down Syndrome Service Register	14	Structured interview with the mothers	(9–36) months	8
Weijerman [52]	2008	2003	Netherlands	Cross‐sectional *controlled*	Children with DS registered on the voluntary national registry Dutch Paediatric Surveillance Unit by paediatricians	182	Questionnaire for the paediatricians	< 5 years	7
Williams [53, 54]	2017 and 2022	2014–2017	United Kingdom	longitudinal	Parents of Children with DS from UK recruited through websites, social media and collaborating health professionals	61	Questionnaire for parents at inclusion, at 6–8 months and at 12 months of infant life	Data collected prospectively. Mean at inclusion: 20 weeks	7

Abbreviations: BF, breastfeeding; cAVSD, complete atrio ventricular septal defect; DS, Down syndrome; SD, standard deviation; VSD, ventricular septal defect.

* In parenthesis sample size with BF data available if different from the total sample size.

^†^ In the case of cross‐sectional studies relying on self‐reported and retrospective data.

^‡^ Only abstract available.

According to the JBI items, most studies had a good sample representativeness (92%) and relevant sampling strategies (84%). The main issue was related to the identification and measurement of breastfeeding status, since 27% of studies were rated as « no » or « unclear » for item 6 and 38% for item 7, which assess whether the methods used to identify and measure the condition are valid and reliable. Except for Pisacane et al. [20], none had an adequate sample size (Table S4).

### 3.3 Population Characteristics

Among studies, the percentage of premature infants in the cohorts varied from 21% to 46%. The percentage of children who received a nasogastric tube was available in 7 studies and ranged from 0% (in a study where nasogastric tube use was an exclusion criterion) to 61% (in children undergoing cardiac surgery). The most reported associated comorbidity was congenital heart disease, described in 13 studies, ranging from 5% to 100% (in studies where heart surgery was an inclusion criterion; Table S1).

All the studies reported an average maternal age at childbirth greater than 30 years. Prevalence of primiparity ranged from 25% to 40% and the percentage of vaginal deliveries ranged from 16% to 85% (Table S2). Of note, a detailed description of the included populations is presented in Table S1 (children) and S2 (mothers).

### 3.4 Primary Outcome

**FIGURE 2:**
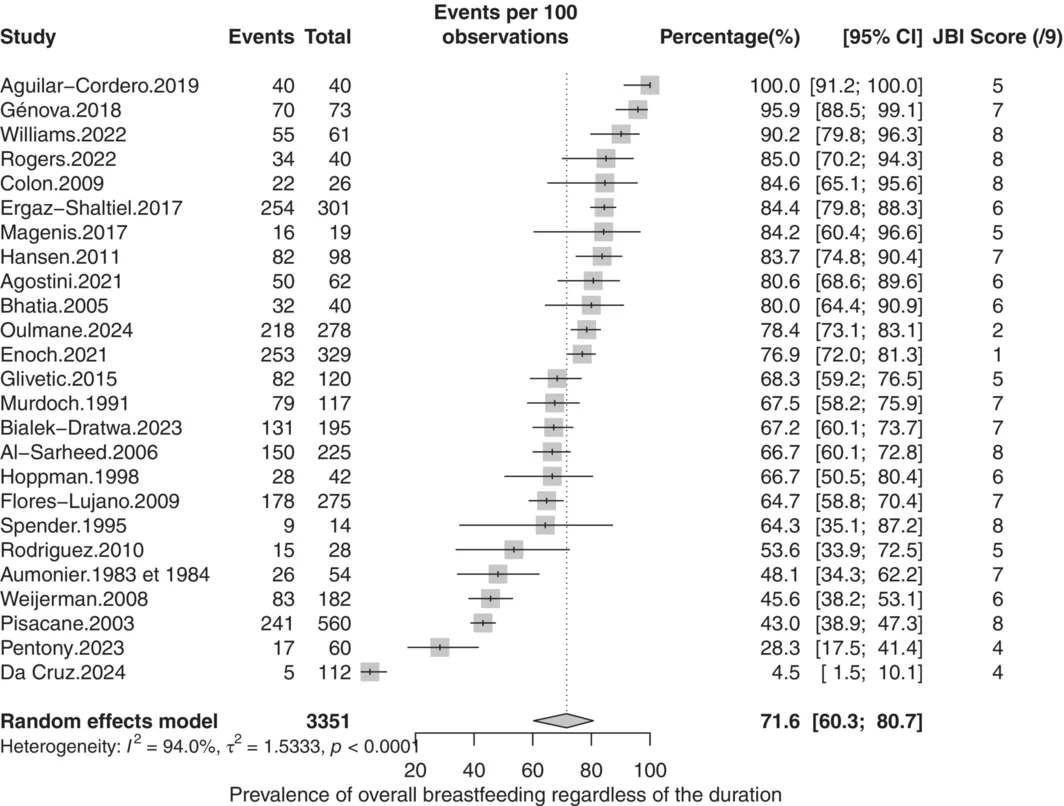
Forest plot: Prevalence of overall breastfeeding regardless of the duration. Individual study prevalence and 95% CIs are shown. The diamond represents the overall pooled prevalence estimated using a random‐effect model. The vertical dashed line indicates the total pooled prevalence.

The primary outcome was the prevalence of breastfeeding regardless of duration. Among the 26 included studies, 25 provided suitable data for the primary outcome, encompassing a total of 3351 participants. The estimated prevalence was 71.6% (95% CI [60.3, 80.7]). A high level of between‐study variability was observed (*I* ^2^ = 94%, τ^2^ = 1.53; Figure 2).

### 3.5 Secondary Outcomes

The estimated prevalence of exclusive breastfeeding, regardless of duration, was 38.4% (95% CI [22.4; 57.3]), with substantial heterogeneity (*I* ^2^ = 90%, 10 studies; Figure S1). The estimated prevalence of partial breastfeeding, regardless of duration, was 24.8% (95% CI [10.5; 48.3]; *I* ^2^ = 89%, 10 studies; Figure S2).

Regarding the estimated prevalence of overall breastfeeding depending on infants' age: the estimated prevalence was 64.5% (95% CI [42.0; 82.1]; *I* ^2^ = 94%) in infants younger than 1 month (11 studies; Figure S3); 67.7% (95% CI [42.5; 85.6]; *I* ^2^ = 93%) in infants between ≥ 1 and < 3 months (6 studies; Figure S4); 51.0% (95% CI [30.9; 70.7]; *I* ^2^ = 96%) in infants between ≥ 3 and < 6 months (5 studies; Figure S5); and 53.4% (95% CI [43.3; 63.2]; *I* ^2^ = 91%) in infants of at least 6 months (9 studies; Figure S6).

Regarding potential factors associated with overall breastfeeding, none of the variables studied, including the promotion of breastfeeding, showed a statistically significant association with the prevalence of overall breastfeeding (Figures S7–S18). Results of meta‐regression, with regression coefficient and corresponding *p*‐value, are summarised in Table 2.

**TABLE 2:** Meta regression of the primary outcome for the 9 variables studied.

Variable	Percentage of premature infants	Percentage of infant's hospitalisation during first year of life	Mean age of mother at delivery (in years)	Percentage of mothers who attended tertiary education	Percentage of primipare mother	Percentage of mother receiving breastfeeding promotion by nursing staff	Level of national breastfeeding promotion policies and programmes in the country according to the WBTI score (/100)	Year of data collection	Percentage of infants with congenital heart disease
	8 studies	7 studies	10 Studies	7 studies.	7 studies	7 studies	19 studies	25 studies	13 studies
Coefficient	−4.94	−4.86	0.24	0.487	−8.52	0.18	0.05	0.02	−0.67
*p*	0.311	0.057	0.109	1.16	0.155	0.899	0.078	0.295	0.601
*I* ^2^ after adjustment	96.3.	98.3	95.4	83.3	86.5	91.9	97.3	97.1	98.1

In children < 5 years at data collection, the estimated prevalence of overall breastfeeding was 77.4% (95% CI [57.6; 89.6]; *I* ^2^ = 93%; 13 studies) and 66.9% (95% CI [56.0; 76.3]; *I* ^2^ = 96%; 6 studies) in children ≥ 5 years at data collection. However, the subgroup analysis did not find a significant difference (*p* = 0.32; Figure S15).

### 3.6 Sensitivity Analysis

The post hoc sensitivity analysis on the primary outcome, excluding 3 studies with very specific population, resulted in an estimated prevalence of overall breastfeeding, regardless of duration, of 73.7% (95% CI [66.8;79.6]; *I* ^2^ = 93%; 22 studies; Figure S1). When excluding studies before 2001, the estimated prevalence of overall breastfeeding, regardless of duration, was 73.4% (95% CI [60.2; 83.5]; *I* ^2^ = 94.9%; 21 studies). The subgroup analysis according to geographical area is provided in Figure S16 and did not find a significant difference (*p* = 0.42).

## 4 Discussion

The present systematic review with meta‐analysis allowed to provide precise data regarding breastfeeding practices in children with Down syndrome in terms of prevalence. We found that while overall breastfeeding was reported in approximately three quarters of children with Down syndrome, exclusive breastfeeding was reported in less than 40% of them. However, no factor potentially associated with breastfeeding was found.

The prevalence found herein is in accordance with previous findings in the general population. A meta‐analysis published in 2017 that investigated breastfeeding practices in the general population estimated a prevalence greater than 80%, regardless of its duration [13]. In addition, the WHO estimated that for the 2015–2022 period, the global prevalence of exclusive breastfeeding in infants aged 0–6 months was 48% [55]. The 65th World Health Assembly developed, in 2012, a global initiative to increase the prevalence of exclusive breastfeeding [56], which was 38% at the time. Following the leadership of the WHO and UNICEF, the Global Breastfeeding Collective has defined an ambitious objective, reaching a 70% prevalence by 2030.

While our findings consolidate the information regarding the breastfeeding prevalence in infants with Down syndrome, the broad main criterion used in our analysis, which allowed for the collection of a large amount of data, contributed to the heterogeneity of the results. The prevalence of ‘overall’ breastfeeding herein encompasses both exclusive, partial or unspecified breastfeeding, and the data were collected at various postpartum intervals across studies, ranging from birth to 6 months.

Regarding between‐study variability, it can be attributed to both variations in the population samples due to the broad eligibility criteria and to various methodologies with limitations in the quality of some studies.

**FIGURE 3:**
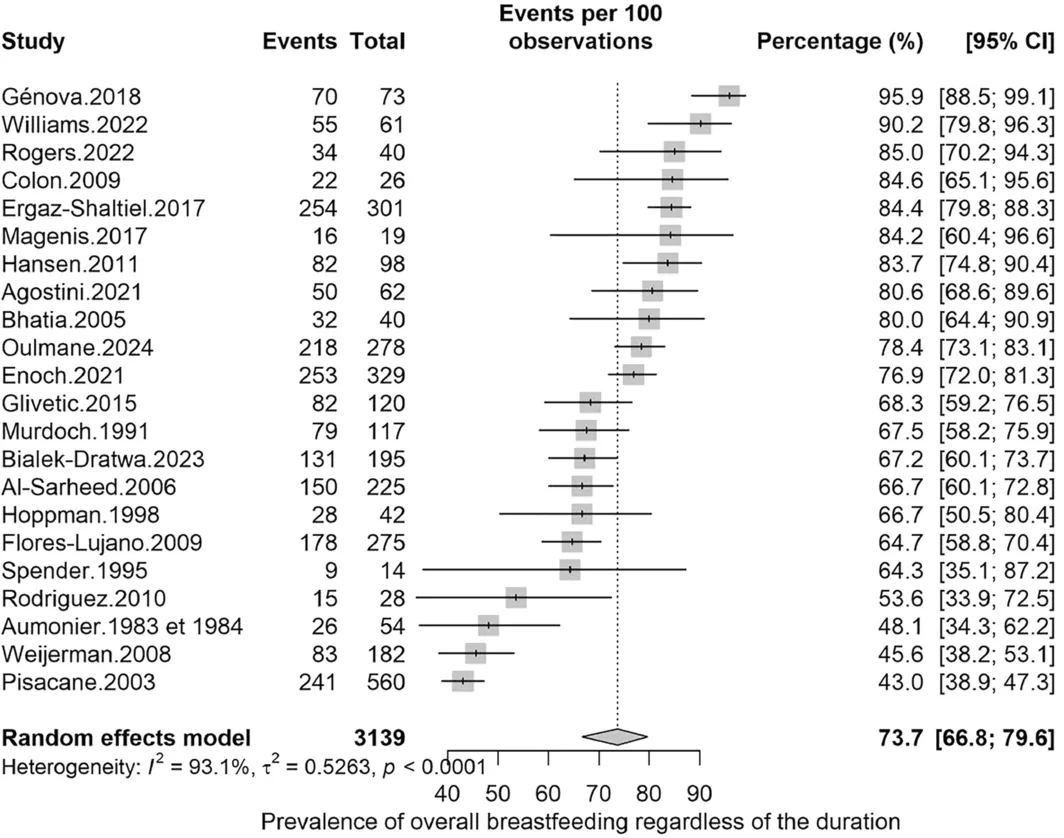
Forest plot: Prevalence of overall breastfeeding regardless of the duration removing Da Cruz Alfaro [28], Pentony [29] and Cordero [30]. Individual study prevalence and 95% CIs are shown. The diamond represents the overall pooled prevalence estimated using a random‐effect model. The vertical dashed line indicates the total pooled prevalence. *Exclusion of study conducted before 2001*: Excluding studies in which data were collected before 2001, the meta‐analysis estimated a prevalence of overall breastfeeding regardless of the duration at: 73.43% (IC95% 60.19–83.48), *I* ^2^ = 94.9% tau^2^ = 1.84 (*n* = 21 studies).

The population samples varied regarding socioeconomic status and country of the mothers as well as regarding infants' initial state of health and comorbidities. This heterogeneity among the present population may be related to the widely varying objectives of the included study and may have led to a selection bias affecting the findings. This variability in the populations studied also reflects the wide diversity of healthcare settings, stemming from differences in study population recruitment, with some populations being followed on an outpatient basis and others in hospital. However, the sensitivity analysis removing three studies with specific populations found a similar prevalence of overall breastfeeding and did not fully account for the observed heterogeneity (Figure 3).

Regarding the heterogeneity attributed to methodological variations among studies, it was related to the outcome definitions and data collection methods. The majority of studies lacked precise definitions of exclusive breastfeeding and the categorisation varied across the included studies; full breastfeeding interpreted as exclusive breastfeeding that could, in some cases, include predominant breastfeeding. Moreover, there was a limited number of studies that provided information on the type of breastfeeding or on its duration; 10 studies performed a precise distinction between exclusive and partial breastfeeding, and 5 studies reported the duration. Regarding breastfeeding assessment, it was not standardised; most of the included studies used self‐reported data in cross‐sectional formats and, most often, the assessment was based on questionnaires given to parents. In 6 studies, parents were interviewed, which might have influenced the responses due to the presence of the interviewer, potentially introducing a social desirability bias. Five studies relied on the analysis of medical records, assuming they were accurately completed beforehand. Overall, these designs and methods of assessment have probably led to measurement error and recall bias. Recall bias is likely to increase with the child's age, as older data are harder to recall accurately; however, subgroup analysis according to children's age at data collection did not reveal significant differences for the primary outcome. We acknowledge this lack of standardisation in the definition of breastfeeding across most studies. Although we attempted to apply the World Health Organisation (WHO) criteria for exclusive and partial breastfeeding, the assessment methods and categorisation of exclusive breastfeeding varied considerably among the included studies.

These limitations – reflected by the loss of JBI scores on Items 6 and 7 for many studies‐ underscore the need for future research using more rigorous and standardised methodologies. We encourage researchers to refer to the WHO definition and measurement methods of breastfeeding published in 2021 [57] and to promote the use of current‐status questions, based on recall of the very recent past.

In secondary objectives, the present meta‐regression did not find any statistically significant variable associated with breastfeeding.

Previous reviews investigated the potential influential factors on breastfeeding practices in infants with Down syndrome; however, no conclusive findings have been made given the limited published data [58]. An interesting finding herein was that breastfeeding promotion by healthcare professionals was not an influential factor. Of note, there was no standardised definition for breastfeeding promotion, which ranged from comprehensive breastfeeding education to simple encouragement. According to a meta‐analysis in the general population, educational and supportive interventions prolonged the duration of exclusive breastfeeding, but the type of breastfeeding promotion could be related to its effectiveness. While interventions that combined group sessions, individual coaching, and telephone follow‐up encompassed the prenatal and postnatal periods and included face‐to‐face and indirect contact were effective [59]; the educational methods limited to brochures and videos have not been found to be statistically effective [60]. In addition, Lewis et al. [16] also highlighted the value of peer support for young mothers, an effective approach in promoting initiation of breastfeeding. It is important to note that in the general population, implementation of the 10 Baby‐friendly Hospital Initiative (BFHI) conditions has been shown to significantly improve breastfeeding rates and duration [61], representing the most effective intervention to improve overall breastfeeding rates [62].

In addition, due to the limited number of studies included in our meta‐regression, conclusions regarding the barriers and effect of variables on breastfeeding prevalence cannot be drawn with certainty. For instance, regarding promotion of breastfeeding by healthcare professionals, the meta‐regression probably lacked statistical power since only seven studies had sufficient data and were included in this analysis.

It has been previously reported that mothers can experience psychological shock when welcoming an infant with Down syndrome [63], and that frequent hospitalisations of these infants disrupt the mother‐baby bond, which can have an impact on the initiation and maintenance of breastfeeding [58]. The present meta‐regression revealed no statistically significant effect of the proportion of infants hospitalised during the first year of life on breastfeeding rates. However, the included studies did not provide detailed information on hospitalisation type and generally applied a broad classification of ‘at least one hospitalisation’. This lack of precision limits the interpretability of the results, as it prevents distinction between prolonged and brief hospital stays, or between single and repeated hospitalisations during the first year of life, which may influence breastfeeding outcomes.

The present findings should be taken with caution. The non‐significant results likely reflect a lack of statistical power resulting from the small number of studies included in some meta‐regressions. The identification of factors influencing breastfeeding requires to investigation in infants with Down syndrome in order to develop appropriate recommendations and subsequent guidelines as well as effective interventions.

While the present meta‐regression did not find any statistically significant variable associated with breastfeeding, previous systematic reviews have reported numerous challenges encountered by mothers of infants with Down syndrome in initiating and sustaining breastfeeding [17, 19, 58]. The studies included in the meta‐analysis also mentioned several specific barriers in breastfeeding of infants with Down syndrome (Table S7). Herein, infants' medical conditions were highlighted, including hypotonia, congenital malformations such as cardiac or gastrointestinal disorders, and abnormal oropharyngeal structures, consistent with previous findings [18, 64]. In addition, across the included studies, healthcare professionals were often reported as not providing adequate information or support for breastfeeding; notably, some professionals were even described as discouraging it and some professionals incorrectly advised that mothers would not be able to breastfeed their child due to their Down syndrome diagnosis [38]. More than half of mothers included in the study by Pisacane et al. [20] reported that they did not receive the necessary support, and Williams et al. [53] found that a quarter of mothers ‘wanted increased help with feeding’. Additionally, Aumonier et al. [33] highlighted that some mothers felt discouraged by the ‘lukewarm’ and ‘casual’ attitude of the hospital staff, which impacted their perseverance in breastfeeding.

## 5 Conclusion and Implications of the Results

In the present meta‐analysis, the estimated prevalence of overall breastfeeding in infants with Down syndrome was approximately 75%, similar to the prevalence reported in the general population. However, these findings should be interpreted with caution due to methodological variability and substantial heterogeneity across studies. They highlight the need for future research on breastfeeding practices in Down syndrome using standardised measurements, as recommended by the WHO guidelines. Establishing such standardisation would be a necessary prerequisite before assessing breastfeeding barriers and facilitators and then would allow to improve healthcare professionals' knowledge as well as their ability to provide support for breastfeeding.

## Author Contributions

**Mélina Balland:** investigation, writing – original draft, writing – review and editing, data curation, methodology. **Julia Prillard:** investigation, writing – original draft, writing – review and editing, data curation, methodology. **Amélie Zelmar:** supervision, writing – review and editing, project administration. **Caroline Giroudon:** conceptualization, supervision, writing – review and editing, resources. **Damien Sanlaville:** supervision, conceptualization, writing – review and editing, project administration. **Mikail Nourredine:** supervision, formal analysis, validation, methodology, writing – review and editing, software, project administration, visualization. **Irène Loras‐Duclaux:** conceptualization, writing – review and editing, project administration, supervision.

## Funding

The authors have nothing to report.

## Conflicts of Interest

The authors declare no conflicts of interest.

## Supporting information


**Figure S1:** Forest plot: prevalence of exclusive breastfeeding regardless of the duration.
**Figure S2:** Forest plot: prevalence of partial breastfeeding regardless of the duration.
**Figure S3:** Forest plot: prevalence of overall breastfeeding from birth to 1 month.
**Figure S4:** Forest plot: prevalence of overall breastfeeding from 1 to 3 months.
**Figure S5:** Forest plot: prevalence of overall breastfeeding from 3 to 6 months.
**Figure S6:** Forest plot: prevalence of overall breastfeeding at 6 months and over.
**Figure S7:** Meta regression of the primary outcome on the percentage of premature infants. 8 studies. The meta‐regression found no effect of the percentage of children born pre‐term on the percentage of breastfeeding (*p* = 0.311. coefficient = −4.94). After adjustment for the percentage of pre‐term children the estimated *I* ^2^ was 96.3.
**Figure S8:** Meta regression of the primary outcome on the percentage of infant's hospitalisation during first year of life. 7 studies. The meta‐regression found no effect of the percentage of hospitalisation of the child during the first year on the percentage of breastfeeding (*p* = 0.057. coefficient = −4.86). After adjustment the estimated *I* ^2^ was 98.3.
**Figure S9:** Meta regression of the primary outcome on the mean age of mother at delivery (in years). 10 studies. The meta‐regression found no effect of the mean maternal age at delivery in the study on the percentage of breastfeeding (*p* = 0.109. coefficient = 0.24). After adjustment the estimated *I* ^2^ was 95.4.
**Figure S10:** Meta regression of the primary outcome on the percentage of mothers who attended tertiary education (i.e., pursue studies after high school diploma or equivalent). 7 studies. The meta‐regression found no effect of the mother's education on the percentage of breastfeeding (*p* = 0.487. coefficient = 1.16). After adjustment the estimated *I* ^2^ was 83.3.
**Figure S11:** Meta regression of the primary outcome on the percentage of primipare mother. 7 studies. The meta‐regression found no effect of the percentage of primiparous women in the study on the percentage of breastfeeding (*p* = 0.155. coefficient = −8.52). After adjustment the estimated *I* ^2^ was 86.5.
**Figure S12:** Meta regression of the primary outcome on the percentage of mother receiving breastfeeding promotion by nursing staff. 7 studies. The meta‐regression found no effect of the percentage of women having received breastfeeding promotion on the percentage of breastfeeding (*p* = 0.899. coefficient = −0.18). After adjustment the estimated *I* ^2^ was 91.9.
**Figure S13:** Meta regression of the primary outcome on the level of national breastfeeding promotion policies and programmes in the country according to the WBTI score (/100). 19 studies. The meta‐regression found no effect of the national WBTI score on the percentage of breastfeeding (*p* = 0.078. coefficient = −0.05). After adjustment, the estimated *I* ^2^ was 97.3.
**Figure S14:** Meta regression of the primary outcome on the year of data collection (i.e., years of the end of data collection or data publication if not specified). 25 studies. The meta‐regression found no effect of the year of data collection on the percentage of breastfeeding (*p* = 0.295. coefficient = 0.02). After adjustment, the estimated *I* ^2^ was 97.1.
**Figure S15:** Forest plot of the primary outcome according to the age of the children with Down syndrome at the time of data collection (< 5 years vs. > 5 years).
**Figure S16:** Forest plot of the primary outcome according to the Geographic zone. This sub‐group analysis is post hoc.
**Figure S17:** Forest plot of the primary outcome according to the Country. This sub‐group analysis is post hoc.
**Figure S18:** Meta regression of the primary outcome on the percentage of infants with congenital heart disease. 13 studies. The meta‐regression did not find an effect of the percentage of infants with congenital heart disease on the percentage of breastfeeding (*p* = 0.601, coefficient = −0.67) After adjustment the estimated *I* ^2^ was 98.

**Table S1:** Infant characteristics in the included studies.
**Table S2:** Maternal characteristics in the included studies.
**Table S3:** Full search strategy and syntax.
**Table S4:** Score JBI: Critical Appraisal Checklist for Prevalence studies from the Joanna BRIGGS institute (/9).
**Table S5:** Excluded studies on full text and reason for exclusion.
**Table S6:** Check list PRISMA.
**Table S7:** Breastfeeding difficulties and reasons for withdrawal reported in studies.

## Data Availability

The data tables supporting the findings of this study are openly available at https://osf.io/xcmbq/.
