# Supplementary material for: Prevalence of Breastfeeding in Infants With Down Syndrome: A Systematic Review and Meta‐Analysis
Source: Acta Paediatr. 2026 Jul 3;115(10):2063–77. doi: 10.1111/apa.70628 (PMC13552598; doi:10.1111/apa.70628)
Supplement: Supplementary file 1 [file APA-115-2063-s001.docx]

**Supplemental tables**

| **Infant characteristics** | | | | | | | | | |
| --- | --- | --- | --- | --- | --- | --- | --- | --- | --- |
| First author | Publication year | Gender  (% male) | Term  (% preterm) | Associated pathology | | Hospitalisation in first year  (% at less 1 hospitalisation in 1^th^ year) | Time of diagnosis  (% prenatal diagnosis) | Feeding difficulties | |
|  |  |  |  | Heart defect | Other pathologies |  |  | RequiringNGT | Any other difficulties |
| Agostini^44^ | 2021 | 54.8% | 0% ^†^ | 100% ^‡^ | Na | Na | Na | Na | "most" difficulty of sucking |
| Aguilar-Cordero^30^ | 2019 | Na | 22.50% | Na | Na | Na | Na | Na | good BF: 25%; regular BF: 30%; bad BF: 45%; poor taking of the breast and bad posture: 60% |
| Al-Sarheed^45^ | 2005 | Na | Na | Na | Na | Na | Na | Na |  |
| Aumonier^46,47^ | 1984 and 1983 | 65% | Na | 47% | respiratory infections: 79% | Na | Na | 27% | bad sucking ability: 47% |
| Bhatia^48^ | 2005 | 65% | 35% | 5% (VSD) | asphyxia: 12.5%; seizures: 12.5% | Na | Na | Na |  |
| Bialek-Dratwa^49^ | 2023 | 49.8% | Na | Na | hypothyroidism: 35.9%; lactose intolerance: 9.7%; celiac disease: 3.6%;oesophageal or pharyngeal dysphagia: 6.2% |  |  | 0% ^‡^ |  |
| Colon^50^ | 2009 | 46.2% | 46.20% | 53.8% had associated health conditions: most heart, respiratory or gastrointestinal | | Na | 38.50% | Na |  |
| Da Cruz Alfaro^28^ | 2024 | 53.6% | 46.43% | 58% | duodenal atresia: 10%;oesophageal atresia: 4%; anal imperforation: 3%; pyelectasis: 4%; hydrocele: 3% | 100% (neonatal intensive care unit) | 27.68% | 56.2% | parenteral alimentation: 11.6% |
| Enoch^43^ | 2021 | Na | Na | Na | Na | Na | Na | Na |  |
| Ergaz-Shaltiel^51^ ; Padilla ^52^ | 2017 | 55% | 21.39% | 79.6% | gastro-intestinal malformations: 6.2% | 35% (neonatal intensive care unit) | N | 11.6% | gastrostomia: 2% |
| Flores-Lujano^53^ | 2009 | 52.4% | Na | 23.2% |  | 15.6% hospitalisation for infection | Na | Na |  |
| Génova^54^ | 2018 | 54.8% | 38.40% | 45.2% | associated health condition: 67.1% | 65.80% | 28.80% | 32.9% | poor weight increase in the first 6 months: 21.9% |
| Glivetic^55^ | 2015 | 53.3% | Na | 5% | Na | Na | Na | Na |  |
| Hansen ^56^ | 2011 | Na | Na | Na | Na | Na | Na | Na |  |
| Hopman^57^ | 1998 | 50% | Na | Na | Na | Na | Na | Na |  |
| Magenis^21^ | 2017 | 68.4% | Na | Na | moderate disabilities: 68.4%; severe disabilities: 31.6% (based on ICF classification) | Na | Na | Na | suction problems: 30.7% |
| Murdoch ^58^ | 1991 | Na | Na | Na | Na | 41% (special care nurseries) | Na | Na |  |
| Oliveira ^59^ | 2010 | 53.6% | Na | Na | Na | Na | Na | Na |  |
| Oulmane^60^ | 2024 | 57.3% | Na | 47% | digestive malformation: 7%; renal and urinary tract anomalies: 2.4%; ocular defects: 70% |  | Na | Na |  |
| Pentony^29^ | 2023 | Na | Na | 100%^‡^ | Na | Na | Na | 62% | calories supplementation before cardiac surgery: 85% |
| Pisacane^20^ | 2003 | 51% | Na | 35% | gastrointestinal malformations requiring surgery: 14%; jaundice: 21% | 44% (neonatal unit) | Na | Na |  |
| Rodriguez Martinez^61^ | 2010 | Na | Na | Na | coeliac disease: 100% (inclusion criteria); thyroid pathology: 35% | Na | Na | Na |  |
| Rogers^62^ | 2022 | 55% | Na | Na | Na | Na | Na | Na |  |
| Spender^63^ | 1996 | 42% | Na | 14% | Na | Na | Na | Na | mothers asking for advice about child's feeding problems: 91% |
| Weijerman^64^ | 2008 | 54% | Na | 55% | duodenal atresia: 2%; Hirschsprung: 1%; congenital diaphragmatic hernia: 1%; pyloric stenosis:1%; congenital cataract: 1%; hydronephrosis: 1% | 86% | Na | Na |  |
| Williams ^41,65^ | 2017 and 2022 | Na | 21% | Na | Na | 56% (special care baby unit) | 25% | 51% | admissions in special care unit due to problems with feeding: 29% of the admission |

DS: Down syndrome; NGT: nasogastric tube; VSD: ventricular septal defect

^†^exclusion criteria

^‡^inclusion criteria

Table S1- Infant characteristics in the included studies

| **Maternal characteristics** | | | | | | | |
| --- | --- | --- | --- | --- | --- | --- | --- |
| First author | Publication year | Age at delivery:  mean or median ±DS (range) | Parity  (% primiparous) | Mode of delivery  (% vaginal delivery) | Prenataly wish to BF (%) | Mother's socio-demographic level: | |
|  |  |  |  |  |  | Education level (% mothers who attended tertiary education) | Details about socio-professional category |
| Agostini^44^ | 2021 | Na | Na | Na | Na | Na | 88.7% had family income up to three minimum wages |
| Aguilar-Cordero^30^ | 2019 | Mean: 31.9± 4.58 | Na | 50% | Na | 42.5% |  |
| Al-Sarheed^45^ | 2005 | Na | Na | Na | Na | Na | 18.7% were illiterate;76.4% were not working |
| Aumonier^42,43^ | 1984 and 1983 | Mean: 30.1 | 29.5% | Na | 47.5% | Na | “they were evenly distributed across the socioeconomic groups” |
| Bhatia^48^ | 2005 | 17.5% were > 30 years at the time of delivery | 25% | 85% | Na | 50% | 90% were housewives;40% of families had monthly income below 5000 Rupees |
| Bialek-Dratwa^49^ | 2023 | Na | Na | Na | Na | 60.5% |  |
| Colon^50^ | 2009 | Mean: 33 (23-45)^†^ | 30.80% | Na | Na | 57.7% |  |
| Da Cruz Alfaro^28^ | 2024 | 71.4% were >35 years at the time of delivery | Na | 16.08% | Na | Na |  |
| Enoch^43^ | 2021 | Na | Na | Na | 97.5% | Na |  |
| Ergaz-Shaltiel^51^ ; Padilla^52^ | 2017 | Mean: 35.6± 7.4 (17-50) | Na | 78% | Na | Na |  |
| Flores-Lujano^53^ | 2009 | Median: 33 | 29.50% | Na | Na | Na | 72% low socio-economic level using Bronfman criteria |
| Génova^54^ | 2018 | Median: 37 (23-47) | Na | 52.10% | 100% | 87.7% | 60.3% of high, 37% of middle and 2.7% of low socioeconomic status (according to CASEN 2013) |
| Glivetic^55^ | 2015 | Mean: 32.6 | 28.3% | 67.5% |  | Na |  |
| Hansen ^56^ | 2011 | Na | Na | Na | Na | Na | 87% with annual household income ≥$50,000 |
| Hopman^57^ | 1998 | Na | Na | Na | Na | Na |  |
| Magenis^21^ | 2017 | Na | Na | Na | Na | Na | 68.4% socio-economic level C-D (according to CCEB with A being the richest stratum of society E and the poorest) |
| Murdoch^58^ | 1991 | Na | Na | Na | Na | Na |  |
| Oliveira^59^ | 2010 | Na | Na | Na | Na | Na |  |
| Oulmane^60^ | 2024 | Mean: 36.0±6.5 | Na | Na | Na | 16.3% | 21.4% had paid employment |
| Pentony^29^ | 2023 | Na | Na | Na | Na | Na |  |
| Pisacane^20^ | 2003 | Na | Na | 59.6% | Na | Na |  |
| Rodriguez Martinez^61^ | 2010 | Na | Na | Na | Na | Na |  |
| Rogers^62^ | 2022 | Na | Na | Na | Na | 67.5% | 75% with weekly household income ≤ £350 |
| Spender^63^ | 1996 | Na | Na | Na | Na | Na |  |
| Weijerman^64^ | 2008 | Mean: 33.6 | 40% | Na | Na | Na |  |
| Williams^41,65^ | 2017 and 2022 | Mean:37 † | 31% | 63% | Na | Na |  |

BF: breastfeeding; DS: Down syndrome; SD: standard deviation; CCEB: Critério de ClassificaçãoEconômicaBrasil.

^†^Maternal age at data collection (data collection up to one year postpartum)

Table S2- Maternal characteristics in the included studies

| **Database** | **Algorithm** |
| --- | --- |
| Pubmed | (("Breast Feeding"[Mesh] OR "Feeding Behavior"[Mesh]) OR (Breast*[Tiab] AND Feeding*[Tiab]) OR Breastfe*[Tiab] OR "Breast Fed*"[Tiab] OR ((Milk*[Tiab] OR feeding*[Tiab]) AND (shar*[Tiab] OR human*[Tiab] OR mother*[Tiab] OR mom[Tiab] OR moms[Tiab] OR parent*[Tiab] OR behavior*[Tiab])) OR lactation*[Tiab] OR "Wet Nursing*"[Tiab]) AND ("Down Syndrome"[Mesh] OR (Down*[Tiab] AND Syndrome*[Tiab]) OR Mongolism*[Tiab] OR Trisom*[Tiab] OR "47.XY.+21"[Tiab] OR "47.XX.+21"[Tiab]) AND (English[lang] OR French[lang] OR Spanish[lang]) |
| WoS | ((Breast* AND Feeding*) OR Breastfe* OR "Breast Fed*" OR ((Milk* OR feeding*) AND (shar* OR human* OR mother* OR mom OR moms OR parent* OR behavior*)) OR lactation* OR "Wet Nursing*") (Topic) And ((Down* AND Syndrome*) OR Mongolism* OR Trisom* OR "47.XY.+21" OR "47.XX.+21") (Topic) And English OR French OR Spanish (Language) |
| Embase | (breast*:ti.ab.kw AND feeding*:ti.ab.kw OR breastfe*:ti.ab.kw OR 'breast fed*':ti.ab.kw OR ((milk*:ti.ab.kw OR feeding*:ti.ab.kw) AND (shar*:ti.ab.kw OR human*:ti.ab.kw OR mother*:ti.ab.kw OR mom:ti.ab.kw OR moms:ti.ab.kw OR parent*:ti.ab.kw OR behavior*:ti.ab.kw)) OR lactation*:ti.ab.kw OR 'wet nursing*':ti.ab.kw) AND (down*:ti.ab.kw AND syndrome*:ti.ab.kw OR mongolism*:ti.ab.kw OR trisom*:ti.ab.kw OR '47.xy.+21':ti.ab.kw OR '47.xx.+21':ti.ab.kw) AND (english:la OR french:la OR spanish:la) |
| Cochrane Library | (((Breast* AND Feeding*) OR Breastfe* OR (Breast* NEXT Fed*) OR ((Milk* OR feeding*) AND (shar* OR human* OR mother* OR mom OR moms OR parent* OR behavior*)) OR lactation* OR (Wet* NEXT Nursing*))):ti.ab.kw AND (((Down* AND Syndrome*) OR Mongolism* OR Trisom* OR "47.XY.+21" OR "47.XX.+21")):ti.ab.kw" (Word variations have been searched) |
| SciELO | (((Breast* AND Feeding*) OR Breastfe* OR "Breast Fed*" OR ((Milk* OR feeding*) AND (shar* OR human* OR mother* OR mom OR moms OR parent* OR behavior*)) OR lactation* OR "Wet Nursing*")) AND (((Down* AND Syndrome*) OR Mongolism* OR Trisom* OR "47.XY.+21" OR "47.XX.+21")) |
| CINAHL | (MH ("Breast Feeding+" OR "Eating Behavior+") OR TI ((Breast* AND Feeding*) OR Breastfe* OR "Breast Fed*" OR lactation* OR "Wet Nursing*" OR ((milk* OR feeding*) AND (shar* OR human* OR mother* OR mom OR moms OR parent* OR behavior*))) OR AB ((Breast* AND Feeding*) OR Breastfe* OR "Breast Fed*" OR lactation* OR "Wet Nursing*" OR ((milk* OR feeding*) AND (shar* OR human* OR mother* OR mom OR moms OR parent* OR behavior*))) OR SU ((Breast* AND Feeding*) OR Breastfe* OR "Breast Fed*" OR lactation* OR "Wet Nursing*" OR ((milk* OR feeding*) AND (shar* OR human* OR mother* OR mom OR moms OR parent* OR behavior*)))) AND (MH "Down Syndrome" OR TI ((Down* AND Syndrome*) OR Mongolism* OR Trisom* OR "47.XY.+21" OR "47.XX.+21") OR AB ((Down* AND Syndrome*) OR Mongolism* OR Trisom* OR "47.XY.+21" OR "47.XX.+21") OR SU ((Down* AND Syndrome*) OR Mongolism* OR Trisom* OR "47.XY.+21" OR "47.XX.+21"))  Languages : English OR French OR Spanish. |

Table S3- Full search strategy and syntax

| **First author and date** | **JBI Items** | | | | | | | | |
| --- | --- | --- | --- | --- | --- | --- | --- | --- | --- |
|  | 1 | 2 | 3 | 4 | 5 | 6 | 7 | 8 | 9 |
| Agostini 2021 | Yes | Yes | No | Yes | Yes | Yes | Yes | No | No |
| Aguilar-cordero 2019 | Yes | No | No | No | Yes | Yes | Yes | Yes | No |
| Al-Sarheed 2005 | Yes | Yes | No | Yes | Yes | Yes | Yes | Yes | Yes |
| Aumonier 1983– 1984 | Yes | Yes | No | Yes | No | Yes | Yes | Yes | Yes |
| Bhatia 2005 | Yes | Yes | No | Yes | Yes | Yes | Unc | No | Yes |
| Bialek-Dratwa 2023 | Yes | Yes | No | Yes | No | Yes | Yes | Yes | Yes |
| Colon 2009 | Yes | Yes | No | Yes | Yes | Yes | Yes | Yes | Yes |
| Da Cruz Alfaro 2024 | No | Yes | No | Yes | Yes | No | No | Yes | Yes |
| Enoch 2021 | Yes | No | No | No | No | No | No | No | No |
| Ergaz-Shaltiel 2017 | Yes | Yes | No | No | No | Yes | Yes | Yes | Yes |
| Flores Lujano 2009 | Yes | Yes | No | Yes | Yes | Yes | Yes | Yes | No |
| Genova 2018 | Yes | Yes | No | Yes | Yes | Yes | Yes | Yes | No |
| Glivetic 2015 | Yes | Yes | No | No | Yes | Unc | No | Yes | Yes |
| Hansen 2011* | Yes | Yes | No | Unc | Unc | Unc | Unc | No | No |
| Hoppman 1998 | Yes | Yes | No | Yes | No | Yes | Yes | Yes | yes |
| Magenis 2017 | Yes | No | No | Yes | Yes | Yes | Yes | Yes | No |
| Murdoch 1991 | Yes | Yes | No | No | Yes | Yes | No | No | Yes |
| Oliveira 2010 | Yes | Yes | No | Yes | Yes | Yes | Yes | Unc | Yes |
| Oulmane 2024 | Yes | Yes | No | Yes | Yes | Yes | Yes | Yes | Yes |
| Pentony 2023* | No | Yes | No | No | Yes | Unc | Unc | Yes | Yes |
| Pisacane 2003 | Yes | Yes | Yes | Yes | Yes | Yes | Yes | Unc | Yes |
| Rogers 2022 | Yes | Yes | No | Yes | Yes | Yes | Yes | Yes | Yes |
| Rodriguez 2010 | Yes | Yes | No | Yes | Yes | No | No | Unc | Yes |
| Spender 1996 | Yes | No | No | Yes | Yes | Unc | Unc | Yes | Unc |
| Williams 2022 | Yes | Yes | No | Yes | Yes | Yes | Yes | Yes | Yes |
| Wejerman 2008 | Yes | Yes | No | No | Yes | Yes | Unc | Yes | Yes |

In this table. « Unc » denotes « unclear ». Articles marked with an asterisk (*) were available only as abstracts so the JBI score was calculated based on the abstract alone.

Table S4-Score JBI: Critical Appraisal Checklist for Prevalence studies from the Joanna BRIGGS institute (/9).

JBI Items:

1) Was the sample frame appropriate to address the target population ?

2) Were study participants sampled in an appropriate way? (YES for probabilistic sampling versus non probabilistic sampling)

3) Was the sample size adequate? (YES for Sample size estimated before the study and attained OR Sample size > 384 participants)

4) Were the study subjects and the setting described in detail ?

5) Was the data analysis conducted with sufficient coverage of the identified sample? YES if all participants were included in the final analysis

6) Were valid method sused for the identification of the condition?

7) Was the condition measured in a standard and reliable way for all participants?

8) Was there appropriate statistical analysis ?YES if the numerator and denominator for estimating prevalence was clearly reported instead of only % and sample size"

9) Was the response rate adequate. and if not. was the low response rate managed appropriately?

| **Authors** | **Title** | **Year** | **Reason for exclusion** |
| --- | --- | --- | --- |
| Amorim | Amamentaçãoemcrianças com síndrome de Down: apercepçãodasmãessobre a atuação dos profïssionais de saúde | 1999 | Wrong language |
| Anil. M.A. | Feeding and swallowing difficulties in children with Down syndrome. | 2019 | Wrong outcomes |
| Arslan. S.S. | Swallowing Related Problems of Toddlers with Down Syndrome | 2023 | Wrong outcomes |
| Barros da Silva.R. | Breastfeeding Experiences of Mothers of Children with Down Syndrome | 2019 | Wrong study design |
| Brantley. C | Feeding Stressors and Available Resources for Parents of Children with Down Syndrome: A Qualitative Analysis... | 2022 | Wrong study design |
| Brantley. C | Feeding Stressors and Resources Used by Caregivers of Children With Down Syndrome: A Qualitative Analysis | 2023 | Wrong study design |
| Burns. K. | Breastfeeding & Down Syndrome. by Ella Gray Cullen. | 2020 | Wrong outcomes |
| Calvert. S. D. | Dietary adequacy. feeding practices. and eating behavior of children with Down's syndrome | 1976 | Wrong outcomes |
| Carneiro. N. C. R | Oral sucking habits among children with Down syndrome and cerebral palsy | 2017 | Wrong patient population |
| Carter. J.A. | No tragedy to see here... | 2017 | Wrong outcomes |
| Cartwright. A. | Feeding infants with Down's Syndrome: A qualitative study of mothers' experiences. | 2018 | Wrong study design |
| Chandran L. | Breastfeeding: the essential principles. | 2006 | Wrong study design |
| Çiyiltepe. M | Factors affecting eating and swallowing skills in children with DS | 2015 | Notretrieved |
| Cochran. E | Introduction of Complementary Foods for Children with Down Syndrome: Parent and Physician Experiences | 2022 | Wrong outcomes |
| Cochran. E | Recommendations and Outcomes for the Introduction of Complementary Foods for Children With Down Syndrome...American Occupational Therapy Association (AOTA) | 2021 | Wrong outcomes |
| Collins MSR. | Eating behaviour and food choices in children with Down's syndrome. autistic spectrum disorder or cri du chat syndrome and comparison groups of siblings: diet and preventive dentistry. | 2004 | Wrong outcomes |
| Connor. G.O | Preliminary report of healthcare status in a cohort of young children aged 1-5 years with Down syndrome | 2011 | Wrong outcomes |
| Cullen. S. M | Social development and feeding milestones of young Down syndrome children | 1981 | Wrong outcomes |
| Danner. S. C. | Breastfeeding the neurologically impaired infant | 1992 | Wrong outcomes |
| Evangelista. L.G. | Fatoresfacilitadores. Principaisdificuldades e estratégiasempregadas no aleitamentomaterno de bebês com síndrome de Down: umarevisãosistemática | 2019 | Wrong study design |
| Farias de Queiroz. M | Nutritional profile of Down syndrome patients in Pernambuco agreste | 2013 | Wrong language |
| Ferro. É. | Nutritional assessment and the influence of breastfeeding in patients with down syndrome | 2017 | Not retrieved |
| Forchielli. M.L. | Birth Weight: An Identity Card for Metabolic Diseases? | 2023 | Wrong outcomes |
| Giaretta. A.G. | Cuidadonutricional:olharsensível. interdisciplinar. para as famílias de pessoas com Síndrome de Down | 2010 | Wrong language |
| González Jiménez. E | Nursing intervention protocol to help establish affective ties between the newborn infant with Down's syndrome and his/her family | 2012 | Wrong outcomes |
| Hielscher. L | A Scoping Review of the Complementary Feeding Practices and Early Eating Experiences of Children With Down Syndrome | 2023 | Wrong study design |
| Hielscher. L | The lived experiences of new mothers accessing feeding support services for infants with Down syndrome during the COVID-19 pandemic. | 2022 | Wrong study design |
| Isrctn. | Feeding and Autoimmunity in Down's syndrome Evaluation Study (FADES) | 2015 | Wrong study design |
| Jönsson. L. | Mothers of children with down syndrome: A qualitative study of experiences of breastfeeding and breastfeeding support | 2022 | Wrong study design |
| Jory. J. | Red cell folate status among a subset of Canadian children with Down Syndrome post-fortification | 2022 | Wrong studydesign |
| Kuhn. D. E. | Feeding Disorders | 2007 | Wrong outcomes |
| Lakshminarayana. P. | KAP study on mothers of children with Down syndrome | 1991 | Wrong outcomes |
| Lewis. E | Parental experiences of feeding problems in their infants with Down syndrome | 2004 | Wrong study design |
| López M | Motivos del abandonotemprano de la lactancia maternal exclusiva: un problema de saludpública no resueltoen la ciudad de Medellín | 2013 | Wrong study design |
| Madasu. S. | The phenotypical. Clinical and cytological profile of down syndrome children: a hospital based study in telangana | 2022 | Wrong outcomes |
| Magenis. M. L. | Down syndrome and breastfeeding: A systematic review | 2022 | Wrong study design |
| McAndrew. S. | NICU management and outcomes of infants with trisomy 21 without major anomalies | 2018 | Wrong outcomes |
| Mengoni. S | Supporting families of children with down syndrome with feeding and eating: Perspectives from healthcare professionals | 2019 | Not retrieved : Conference Abstract |
| Mengoni. S | Feeding and eating problems in children with down syndrome: A mixed-methods study | 2019 | Not retrieved : Conference Abstract |
| Miki Mizukami. | Investigating factors related to the acquisition of masticatory function in down syndrome children. | 2019 | Wrong outcomes |
| Mortimer. G.L.M. | Feeding and autoimmunity in children with Down syndrome evaluation study (FADES): Evidence of early autoimmunity to insulin | 2023 | Wrong outcomes |
| Hussain. W.S. | Effect of neuromuscular electrical stimulation on dysphagia in children with down syndrome | 2022 | Wrong outcomes |
| O'Neill. K. L. | Child-feeding practices in children with down syndrome and their siblings | 2055 | Wrong outcomes |
| Oliveira. A. C. | Prevalence and determinant factors of malocclusion in children with special needs | 2011 | Wrong patient population |
| Patel. L. | Patterns of Behavior and Medical Comorbidities in down Syndrome | 2020 | Wrong outcomes |
| Pettitt. R.M. | Newborn disorders and nutritional guidance | 2018 | Wrong study design |
| Phillips. C. | Relationship-Based Care for Newborns With Down Syndrome and Endocardial Cushion Defect | 2015 | Wrong outcomes |
| Pipes. P. | Feeding children with Down's syndrome | 1980 | Not retrieved |
| Ramanathan. A. | A study to assess the effectiveness of oro-motor and behavioral approach to reduce tongue thrust in children with down's syndrome | 2016 | Not retrieved : Conference Abstract |
| Randell. D. M. | Preventive dental health practices of non-institutionalized Down syndrome children: a controlled study | 1192 | Wrong outcomes |
| Ravel. A | Feeding problems and gastrointestinal diseases in Down syndrome | 2020 | Wrong outcomes |
| Reyna. B. | Breastfeeding and Down Syndrome: A Comprehensive Guide for Mothers and Medical Professionals | 2020 | Wrong study design |
| Rogers. S.L. | Relationships between feeding problems and eating behaviours in children with and without Down Syndrome. | 2021 | Wrong outcomes |
| Salikhova. K. | Speech disorders in children with down syndrome | 2019 | Wrong outcomes |
| SerelArslan. S. | Swallowing Related Problems of Toddlers with Down Syndrome | 2022 | Wrong outcomes |
| Smith. M. D | Feeding Outcomes in Neonates With Trisomy 21 and Duodenal Atresia | 2019 | Wrong outcomes |
| Sooben. R. | Breastfeeding of newborns with Down's syndrome. | 2015 | Wrong outcomes |
| Sooben. R. | Breastfeeding patterns in infants with Down's syndrome: A literature review. | 2012 | Wrong study design |
| Spinazzi. N. A. | Co-occurring conditions in children with Down syndrome and autism: a retrospective study | 2023 | Wrong outcomes |
| Stanley. M. A | Clinical identification of feeding and swallowing disorders in 0-6 month old infants with Down syndrome | 2019 | Wrong outcomes |
| Surette. V. A. | Observations of feeding practices of US parents of young children with Down syndrome | 223 | Wrong outcomes |
| Thomas. J | ABM Clinical Protocol #16: Breastfeeding the Hypotonic Infant | 2007 | Wrong study design |
| Treider. M. | Is postoperative transanastomotic feeding beneficial in neonates with congenital duodenal obstruction? | 2022 | Wrong patient population |
| Tuohy. M. | Dietary management of chylothorax in congenital heart disease | 2019 | Wrong patient population |
| van Dijk. M | Measuring feeding difficulties in toddlers with Down syndrome | 2018 | Wrong outcomes |
| Wieczorkievicz.A. M. | O processo de amamentação de mulheresmães de criançasportadoras de Síndrome de Down | 2009 | Wrong language |
| Williams. G. | FADES: A birth cohort to understand the mechanisms underlying accelerated onset of autoimmunity in children with down's syndrome | 2019 | Wrong outcomes |
| Wood. S | Asking parents/carers of young children with Down syndrome about specific eating behaviours and feeding practices could lead to more effective tailored support for feeding problems | 2022 | Wrong outcomes |
| Zhen. L. | Can I breastfeed my baby with Down syndrome? A scopingreview | 2021 | Wrong study design |
| by la lecheleague international | Perinatal. Breast-feeding when your baby has hypotonia. congenital heart disease. or Down syndrome. | 1991 | Wrong outcomes |
| Britton. J.R. | Breastfeeding. sensitivity. and Attachment | 2006 | Wrong study design |
| Sacker. A. | Breastfeeding and developmental Delay : findings forms the millennium cohort study. | 2006 | Wrong patient population |
| Jea. A. | Moyamoya syndrome associated with Down Syndrome : outcome after surgical revascularization | 2005 | Wrong outcomes |
| American academy of family physicians. | Patient Information. Caring for your baby with Down syndrome | 2014 | Wrong study design |
| “news” | Birth Issues in Perinatal Care | 2007 | Wrong patient population |
| “MIDIRS Update” | Essentially MIDIRS dec 2014 | 2014 | Not retrieved |
| “MIDIRS Update” | Essentially MIDIRS dec 2012 | 2012 | Not retrieved |
| “World IrNurs “ | Helping mentally handicapped children to eat | 1981 | Not retrieved |
| The association of radical midwives | Midwifery Matters : breastfeed soonest. breastfeed longest | 1999 | Wrong patient population |
| “World of irish nursing” | Feeding the child who has Down syndrome | 1981 | Wrong study design |
| “Midwifery News” | Midwifery News September 2008 | 2008 | Wrong patient population |

Table S5- Excluded studies on full text and reason for exclusion

| **Section and Topic** | **Item** | **Checklist item** | **Location** |
| --- | --- | --- | --- |
| **TITLE** | | |  |
| Title | 1 | Identify the report as a systematic review. | 1 |
| **ABSTRACT** | | |  |
| Abstract | 2 | See the PRISMA 2020 for Abstracts checklist. | 2 |
| **INTRODUCTION** | | |  |
| Rationale | 3 | Describe the rationale for the review in the context of existing knowledge. | 3 |
| Objectives | 4 | Provide an explicit statement of the objective(s) or question(s) the review addresses. | 3 |
| **METHODS** | | |  |
| Eligibility criteria | 5 | Specify the inclusion and exclusion criteria for the review   and how studies were grouped for the syntheses. | 4 |
| Information sources | 6 | Specify all databases. registers. websites. organisations. reference lists and other sources searched or consulted to identify studies. Specify the date when each source was last searched or consulted. | 4-5 |
| Search strategy | 7 | Present the full search strategies for all databases. registers and websites. including any filters and limits used. | 4-5 |
| Selection process | 8 | Specify the methods used to decide whether a study met the inclusion criteria of the review. including how many reviewers screened each record and each report retrieved. whether they worked independently. and if applicable. details of automation tools used in the process. | 5 |
| Data collection process | 9 | Specify the methods used to collect data from reports. including how many reviewers collected data from each report. whether they worked independently. any processes for obtaining or confirming data from study investigators. and if applicable. details of automation tools used in the process. | 5 |
| Data items | 10a | List and define all outcomes for which data were sought. Specify whether all results that were compatible with each outcome domain in each study were sought (e.g. for all measures. time points. analyses). and if not. the methods used to decide which results to collect. | 5-6 |
|  | 10b | List and define all other variables for which data were sought (e.g. participant and intervention characteristics. funding sources). Describe any assumptions made about any missing or unclear information. | 6 |
| Study risk of bias assessment | 11 | Specify the methods used to assess risk of bias in the included studies. including details of the tool(s) used. how many reviewers assessed each study and whether they worked independently. and if applicable. details of automation tools used in the process. | 5 |
| Effect measures | 12 | Specify for each outcome the effect measure(s) (e.g. risk ratio. mean difference) used in the synthesis or presentation of results. | x |
| Synthesis methods | 13a | Describe the processes used to decide which studies were eligible for each synthesis (e.g. tabulating the study intervention characteristics and comparing against the planned groups for each synthesis (item #5)). | 5-6 |
|  | 13b | Describe any methods required to prepare the data for presentation or synthesis. such as handling of missing summary statistics. or data conversions. | 6 |
|  | 13c | Describe any methods used to tabulate or visually display results of individual studies and syntheses. | 6-7 |
|  | 13d | Describe any methods used to synthesise results and provide a rationale for the choice(s). If meta-analysis was performed. describe the model(s). method(s) to identify the presence and extent of statistical heterogeneity. and software package(s) used. | 6-7 |
|  | 13e | Describe any methods used to explore possible causes of heterogeneity among study results (e.g. subgroup analysis. meta-regression). | 7 |
|  | 13f | Describe any sensitivity analyses conducted to assess robustness of the synthesised results. | 7 |
| Reporting bias assessment | 14 | Describe any methods used to assess risk of bias due to missing results in a synthesis (arising from reporting biases). | 7 |
| Certainty assessment | 15 | Describe any methods used to assess certainty (or confidence) in the body of evidence for an outcome. | 7 |
| **RESULTS** | | |  |
| Study selection | 16a | Describe the results of the search and selection process. from the number of records identified in the search to the number of studies included in the review. ideally using a flow diagram. | 8 |
|  | 16b | Cite studies that might appear to meet the inclusion criteria. but which were excluded. and explain why they were excluded. | 8 |
| Study characteristics | 17 | Cite each included study and present its characteristics. | 8 |
| Risk of bias in studies | 18 | Present assessments of risk of bias for each included study. | 8 |
| Results of individual studies | 19 | For all outcomes. present. for each study: (a) summary statistics for each group (where appropriate) and (b) an effect estimate and its precision (e.g. confidence/credible interval). ideally using structured tables or plots. | 9 |
| Results of syntheses | 20a | For each synthesis. briefly summarise the characteristics and risk of bias among contributing studies. | 10 |
|  | 20b | Present results of all statistical syntheses conducted. If meta-analysis was done. present for each the summary estimate and its precision (e.g. confidence/credible interval) and measures of statistical heterogeneity. If comparing groups. describe the direction of the effect. | 9-10 |
|  | 20c | Present results of all investigations of possible causes of heterogeneity among study results. | 10 |
|  | 20d | Present results of all sensitivity analyses conducted to assess the robustness of the synthesised results. | 10 |
| Reporting biases | 21 | Present assessments of risk of bias due to missing results (arising from reporting biases) for each synthesis assessed. | 10 |
| Certainty of evidence | 22 | Present assessments of certainty (or confidence) in the body of evidence for each outcome assessed. | 10 |
| **DISCUSSION** | | |  |
| Discussion | 23a | Provide a general interpretation of the results in the context of other evidence. | 11 |
|  | 23b | Discuss any limitations of the evidence included in the review. | 14-15 |
|  | 23c | Discuss any limitations of the review processes used. | 14 |
|  | 23d | Discuss implications of the results for practice. policy. and future research. | 13 |
| **OTHER INFORMATION** | | |  |
| Registration and protocol | 24a | Provide registration information for the review. including register name and registration number. or state that the review was not registered. | 4 |
|  | 24b | Indicate where the review protocol can be accessed. or state that a protocol was not prepared. | 4 |
|  | 24c | Describe and explain any amendments to information provided at registration or in the protocol. | 7 |
| Support | 25 | Describe sources of financial or non-financial support for the review. and the role of the funders or sponsors in the review. | 16 |
| Competing interests | 26 | Declare any competing interests of review authors. | 16 |
| Availability of data. code and other materials | 27 | Report which of the following are publicly available and where they can be found: template data collection forms; data extracted from included studies; data used for all analyses; analytic code; any other materials used in the review. | 16 |

Table S6- Check list PRISMA

| **Breastfeeding difficulties and reasons for withdrawal** | | | |
| --- | --- | --- | --- |
| First author | Maternal barriers or difficulty to breastfeeding | Child related barriers or difficulty to breastfeeding | Health service barriers to breastfeeding or discouraging nursing staff attitude |
| Agostini^44^ | “some” maternal nervousness causing them not to produce milk. | “most” because of child's tiredness;  “most” because of difficulty of sucking;  “some” hospitalisation soon after birth. | Na |
| Aguilar-Cordero^30^ | “a lot” of breast engorgement. | 60% of poor taking of the breast;  Bad posture with newborn head not aligned with the body. | Na |
| Al-Sarheed^45^ | Na | Na | “some” mothers were discouraged from BF. |
| Aumonier^46,47^ | Inadequate milk supply as the main reason;  6% of others wishing to breast feed antenataly were too ill post-nataly. | Na | 21% whose antenatal preference was not to breastfeed did not feel encouraged by hospital staff to initiate BF;  83% whose antenatal preference was undecided were unaware of any attempt by hospital staff to promote BF;  3% mothers wishing to breastfeed were discouraged by the hospital staff;  26% said the hospital staff made no attempt to encourage BF;  9.8% were not encouraged to express milk to give to their babies in SCU. |
| Bhatia^48^ | Na | Na | Na |
| Bialek-Dratwa^49^ | 27.7% of mother couldn’t BF for medical reasons. | Na | Na |
| Colon^50^ | 61.5% of mother would not breastfeed in public places. | 84.6% of baby sucking problems. | 50.0% of mother not allowed to have their baby with them during the stay in hospital;  30.8% of mother did not received support from hospital personnel. |
| Da Cruz Alfaro^28^ | Na | Na | Na |
| Enoch^43^ | Na | Na | 46% mother had been advised by a healthcare professional that they may not be able to breastfeed their infant purely because of baby having DS;  54% of mother did not receive support they needed. |
| Ergaz-Shaltiel^51^ ; Padilla ^52^ | Na | Na | Na |
| Flores-Lujano^53^ | Na | Na | Na |
| Génova^54^ | 10.2% because of maternal reasons: 5.1% mother disease and 5.1% back to work. | 64.1% because of child factors: 28.2% poor weight increase; 23.1% suction disorder; 5.1% associated disease; 7.7% hungry baby. | 38.5% due to health care factors: 17.9% hospitalisation and 20.5% prescription of formula despite good weight increase. |
| Glivetic^55^ | Na | Na | Na |
| Hansen ^56^ | Na | Na | Only 5% reported that a medical professional provided information about BF an infant with DS and when information was provided, it was often inconsistent. |
| Hopman^57^ | Na | Na | Na |
| Magenis^21^ | 23% because of maternal health;  15.3% because of insufficient milk. | 30.7% because of suction problems;  5.3% because of prematurity. | 15.3% because of early complementary feeding. |
| Murdoch ^58^ | 13% of mother spends only some feeding or none feeding with the baby. | Na | Only 46% of the general practitioner where helpful;  Only 51% of the plunket nurse were helpful. |
| Oliveira ^59^ | Na | Na | Na |
| Oulmane^60^ | Na | Na | Na |
| Pentony^29^ | Na | Na | Na |
| Pisacane^20^ | Among mothers of infants not admitted to the hospital:35% of frustration or depression; 26% of fear of milk insufficiency. | Among infants in neonatal unit: 70% because of illness of the baby.  Among infants not admitted to the hospital: 21% because of sucking difficulty. | Na |
| Rodriguez Martinez^61^ | Na | Na | Na |
| Rogers^62^ | Na | Na | Na |
| Spender^63^ | Na | Na | Na |
| Weijerman^64^ | Na | Na | Na |
| Williams ^41,65^ | At initial questionnaire:  30% of inadequate breastmilk supply;  25% of problems expressing milk;  25% of excessive time expressing milk;  15% of maternal medical problem.  Weaning by choice: 7% at 7 months, 8% at 12 months. | 61% of poor weight gain; 20% of baby losing weight;5% because of lethargy;  44% of poor latch; 2% of low tone;29% of tired 29%. | 17% of mothers did not receive enough help with feeding in hospital/birth centre. |

BF: Breastfeeding; SCU: special care unit

Table S7– Breastfeeding difficulties and reasons for withdrawal reported in studies.
