# Supplementary material for: Prevalence of Breastfeeding in Infants With Down Syndrome: A Systematic Review and Meta‐Analysis
Source: Acta Paediatr. 2026 Jul 3;115(10):2063–77. doi: 10.1111/apa.70628 (PMC13552598; doi:10.1111/apa.70628)
Supplement: Supplementary file 2 [file APA-115-2063-s002.zip › apa70628-sup-0001-DataS1.docx]

# Supplemental figures legends

Figure S1- Forest plot: prevalence of exclusive breastfeeding regardless of the duration.

Individual study prevalence and 95% CIs are shown. The diamond represents the overall pooled prevalence estimated using a random-effect model. The vertical dashed line indicates the total pooled prevalence.

Figure S2- Forest plot: prevalence of partial breastfeeding regardless of the duration.

Individual study prevalence and 95% CIs are shown. The diamond represents the overall pooled prevalence estimated using a random-effect model. The vertical dashed line indicates the total pooled prevalence.

Figure S3- Forest plot: prevalence of overall breastfeeding from birth to 1 month.

Individual study prevalence and 95% CIs are shown. The diamond represents the overall pooled prevalence estimated using a random-effect model. The vertical dashed line indicates the total pooled prevalence.

Figure S4- Forest plot: prevalence of overall breastfeeding from 1 to 3 months.

Individual study prevalence and 95% CIs are shown. The diamond represents the overall pooled prevalence estimated using a random-effect model. The vertical dashed line indicates the total pooled prevalence.

Figure S5- Forest plot: prevalence of overall breastfeeding from 3 to 6 months.

Individual study prevalence and 95% CIs are shown. The diamond represents the overall pooled prevalence estimated using a random-effect model. The vertical dashed line indicates the total pooled prevalence.

Figure S6- Forest plot: prevalence of overall breastfeeding at 6 months and over.

Individual study prevalence and 95% CIs are shown. The diamond represents the overall pooled prevalence estimated using a random-effect model. The vertical dashed line indicates the total pooled prevalence.

Figure S7 - Meta regression of the primary outcome on the percentage of premature infants. 8 studies. The meta-regression found no effect of the percentage of children born pre-term on the percentage of breastfeeding (p=0.311. coefficient =-4.94). After adjustment for the percentage of pre-term children the estimated I² was 96.3.

Figure S8- Meta regression of the primary outcome on the percentage of infant’s hospitalisation during first year of life. 7 studies. The meta-regression found no effect of the percentage of hospitalisation of the child during the first year on the percentage of breastfeeding (p=0.057. coefficient =-4.86). After adjustment the estimated I² was 98.3.

Figure S9- Meta regression of the primary outcome on the mean age of mother at delivery (in years). 10 Studies. The meta-regression found no effect of the mean maternal age at delivery in the study on the percentage of breastfeeding (p=0.109. coefficient =0.24). After adjustment the estimated I² was 95.4.

Figure S10- Meta regression of the primary outcome on the percentage of mothers who attended tertiary education (i.e. pursue studies after high school diploma or equivalent). 7 studies. The meta-regression found no effect of the mother's education on the percentage of breastfeeding (p=0.487. coefficient =1.16). After adjustment the estimated I² was 83.3.

Figure S11- Meta regression of the primary outcome on the percentage of primipare mother. 7 studies. The meta-regression found no effect of the percentage of primiparous women in the study on the percentage of breastfeeding (p=0.155. coefficient =-8.52). After adjustment the estimated I² was 86.5.

Figure S12- Meta regression of the primary outcome on the percentage of mother receiving breastfeeding promotion by nursing staff.7 studies. The meta-regression found no effect of the percentage of women having received breastfeeding promotion on the percentage of breastfeeding (p=0.899. coefficient =-0.18). After adjustment the estimated I² was 91.9.

Figure S13- Meta regression of the primary outcome on the level of national breastfeeding promotion policies and programmes in the country according to the WBTI score (/100). 19 studies. The meta-regression found no effect of the national WBTI score on the percentage of breastfeeding (p=0.078. coefficient =-0.05). After adjustment, the estimated I² was 97.3.

Figure S14- Meta regression of the primary outcome on the year of data collection (i.e. years of the end of data collection or data publication if not specified). 25 studies. The meta-regression found no effect of the year of data collection on the percentage of breastfeeding (p=0.295. coefficient =0.02). After adjustment, the estimated I² was 97.1.

Figure S15- Forest plot of the primary outcome according to the age of the children with Down syndrome at the time of data collection (< 5 years VS > 5 years).

Individual study prevalence and 95% CIs are shown. The diamond represents the overall pooled prevalence estimated using a random-effect model. The vertical dashed line indicates the total pooled prevalence.

Figure S16- Forest plot of the primary outcome according to the Geographic zone. This sub-group analysis is post-hoc.

Individual study prevalence and 95% CIs are shown. The diamond represents the overall pooled prevalence estimated using a random-effect model. The vertical dashed line indicates the total pooled prevalence.

Figure S17- Forest plot of the primary outcome according to the Country. This sub-group analysis is post-hoc.

Individual study prevalence and 95% CIs are shown. The diamond represents the overall pooled prevalence estimated using a random-effect model. The vertical dashed line indicates the total pooled prevalence.

Figure S18- Meta regression of the primary outcome on the percentage of of infants with congenital heart disease. 13 studies. The meta-regression did not find an effect of the percentage of of infants with congenital heart disease on the percentage of breastfeeding (p=0.601, coefficient =-0.67) After adjustment the estimated I² was 98.
