# Supplementary material for: Prevalence of Breastfeeding in Infants With Down Syndrome: A Systematic Review and Meta‐Analysis
Source: Acta Paediatr. 2026 Jul 3;115(10):2063–77. doi: 10.1111/apa.70628 (PMC13552598; doi:10.1111/apa.70628)
Supplement: Supplementary file 2 [file APA-115-2063-s002.zip › apa70628-sup-0002-FigureS1.pdf]

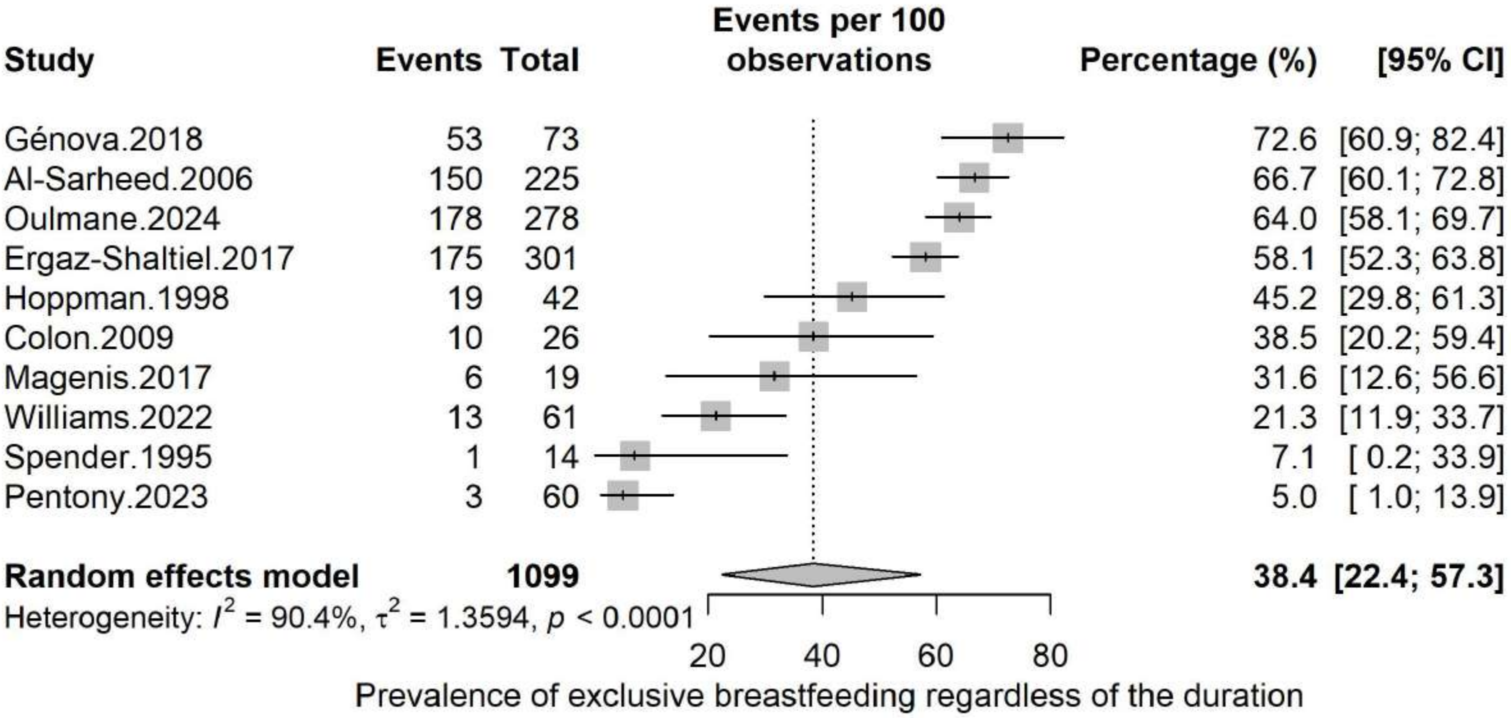
Study
Events
Total
Events per 100 observations
Percentage (%)
[95% CI]
Génova.2018 53 73 72.6 [60.9; 82.4]
Al-Sarheed.2006 150 225 66.7 [60.1; 72.8]
Oulmane.2024 178 278 64.0 [58.1; 69.7]
Ergaz-Shaltiel.2017 175 301 58.1 [52.3; 63.8]
Hoppman.1998 19 42 45.2 [29.8; 61.3]
Colon.2009 10 26 38.5 [20.2; 59.4]
Magenis.2017 6 19 31.6 [12.6; 56.6]
Williams.2022 13 61 21.3 [11.9; 33.7]
Spender.1995 1 14 7.1 [0.2; 33.9]
Pentony.2023 3 60 5.0 [1.0; 13.9]
Random effects model 1099 38.4 [22.4; 57.3]
Heterogeneity: I^2 = 90.4%, τ^2 = 1.3594, p < 0.0001
20
40
60
80
Prevalence of exclusive breastfeeding regardless of the duration
