# Supplementary material for: Prevalence of Breastfeeding in Infants With Down Syndrome: A Systematic Review and Meta‐Analysis
Source: Acta Paediatr. 2026 Jul 3;115(10):2063–77. doi: 10.1111/apa.70628 (PMC13552598; doi:10.1111/apa.70628)
Supplement: Supplementary file 2 [file APA-115-2063-s002.zip › apa70628-sup-0003-FigureS2.pdf]

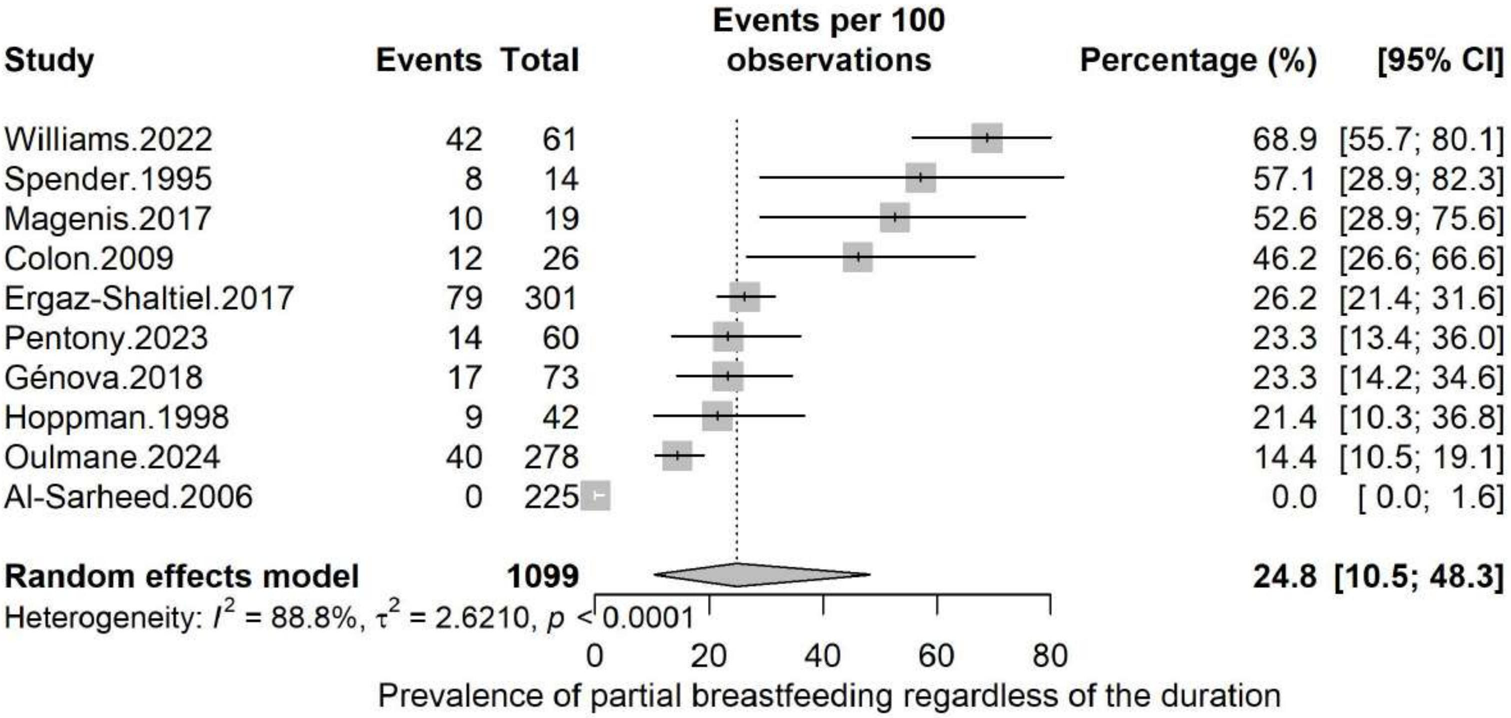
Study
Events
Total
Events per 100 observations
Percentage (%)
[95% CI]
Williams.2022 42 61 68.9 [55.7; 80.1]
Spender.1995 8 14 57.1 [28.9; 82.3]
Magenis.2017 10 19 52.6 [28.9; 75.6]
Colon.2009 12 26 46.2 [26.6; 66.6]
Ergaz-Shaltiel.2017 79 301 26.2 [21.4; 31.6]
Pentony.2023 14 60 23.3 [13.4; 36.0]
Génova.2018 17 73 23.3 [14.2; 34.6]
Hoppman.1998 9 42 21.4 [10.3; 36.8]
Oulmane.2024 40 278 14.4 [10.5; 19.1]
Al-Sarheed.2006 0 225 0.0 [0.0; 1.6]
Random effects model 1099 24.8 [10.5; 48.3]
Heterogeneity: I^2 = 88.8%, τ^2 = 2.6210, p < 0.0001
0
20
40
60
80
Prevalence of partial breastfeeding regardless of the duration
