# Supplementary material for: Prevalence of Breastfeeding in Infants With Down Syndrome: A Systematic Review and Meta‐Analysis
Source: Acta Paediatr. 2026 Jul 3;115(10):2063–77. doi: 10.1111/apa.70628 (PMC13552598; doi:10.1111/apa.70628)
Supplement: Supplementary file 2 [file APA-115-2063-s002.zip › apa70628-sup-0004-FigureS3.pdf]

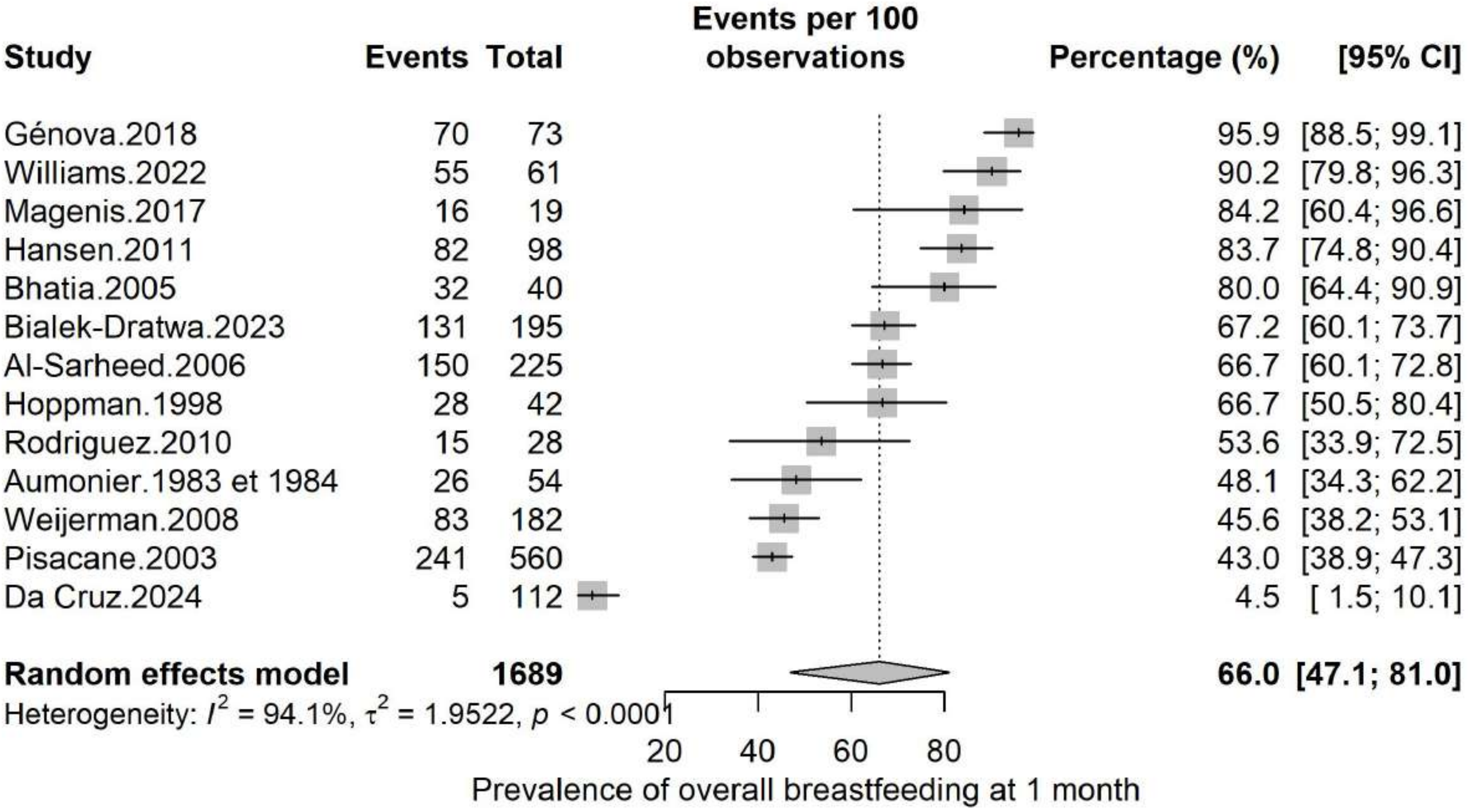
Study
Events
Total
Events per 100 observations
Percentage (%)
[95% CI]
Génova.2018 70 73 95.9 [88.5; 99.1]
Williams.2022 55 61 90.2 [79.8; 96.3]
Magenis.2017 16 19 84.2 [60.4; 96.6]
Hansen.2011 82 98 83.7 [74.8; 90.4]
Bhatia.2005 32 40 80.0 [64.4; 90.9]
Bialek-Dratwa.2023 131 195 67.2 [60.1; 73.7]
Al-Sarheed.2006 150 225 66.7 [60.1; 72.8]
Hoppman.1998 28 42 66.7 [50.5; 80.4]
Rodriguez.2010 15 28 53.6 [33.9; 72.5]
Aumonier.1983 et 1984 26 54 48.1 [34.3; 62.2]
Weijerman.2008 83 182 45.6 [38.2; 53.1]
Pisacane.2003 241 560 43.0 [38.9; 47.3]
Da Cruz.2024 5 112 4.5 [1.5; 10.1]
Random effects model 1689 66.0 [47.1; 81.0]
Heterogeneity: I^2 = 94.1%, τ^2 = 1.9522, p < 0.0001
20
40
60
80
Prevalence of overall breastfeeding at 1 month
