# Supplementary material for: Prevalence of Breastfeeding in Infants With Down Syndrome: A Systematic Review and Meta‐Analysis
Source: Acta Paediatr. 2026 Jul 3;115(10):2063–77. doi: 10.1111/apa.70628 (PMC13552598; doi:10.1111/apa.70628)
Supplement: Supplementary file 2 [file APA-115-2063-s002.zip › apa70628-sup-0005-FigureS4.pdf]

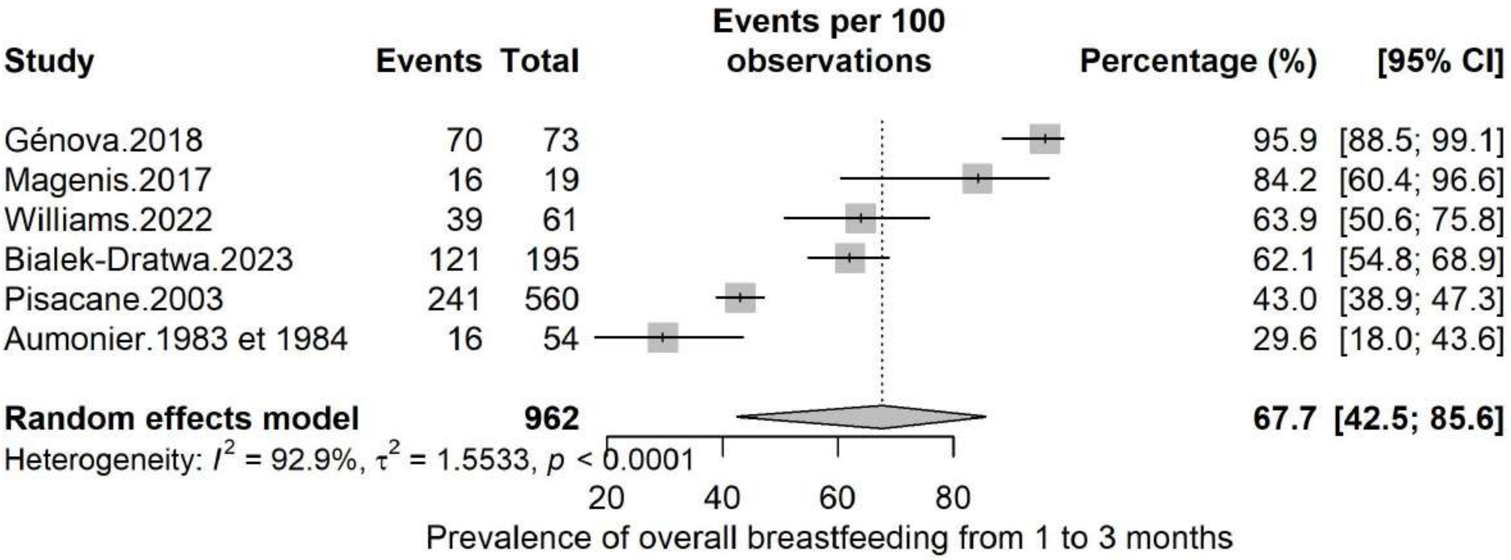
Study
Events
Total
Events per 100 observations
Percentage (%)
[95% CI]
Génova.2018 70 73 95.9 [88.5; 99.1]
Magenis.2017 16 19 84.2 [60.4; 96.6]
Williams.2022 39 61 63.9 [50.6; 75.8]
Bialek-Dratwa.2023 121 195 62.1 [54.8; 68.9]
Pisacane.2003 241 560 43.0 [38.9; 47.3]
Aumonier.1983 et 1984 16 54 29.6 [18.0; 43.6]
Random effects model 962 67.7 [42.5; 85.6]
Heterogeneity: I^2 = 92.9%, τ^2 = 1.5533, p < 0.0001
20
40
60
80
Prevalence of overall breastfeeding from 1 to 3 months
