# Supplementary material for: Prevalence of Breastfeeding in Infants With Down Syndrome: A Systematic Review and Meta‐Analysis
Source: Acta Paediatr. 2026 Jul 3;115(10):2063–77. doi: 10.1111/apa.70628 (PMC13552598; doi:10.1111/apa.70628)
Supplement: Supplementary file 2 [file APA-115-2063-s002.zip › apa70628-sup-0006-FigureS5.pdf]

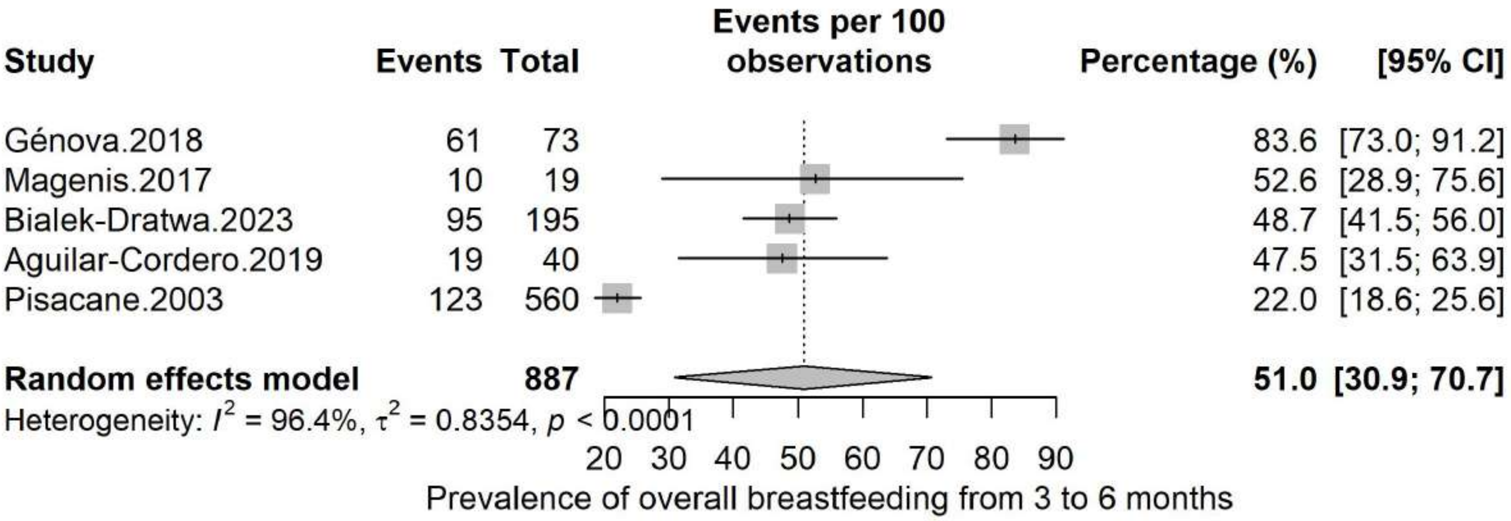
Study
Events
Total
Events per 100 observations
Percentage (%)
[95% CI]
Génova.2018 61 73 83.6 [73.0; 91.2]
Magenis.2017 10 19 52.6 [28.9; 75.6]
Bialek-Dratwa.2023 95 195 48.7 [41.5; 56.0]
Aguilar-Cordero.2019 19 40 47.5 [31.5; 63.9]
Pisacane.2003 123 560 22.0 [18.6; 25.6]
Random effects model 887 51.0 [30.9; 70.7]
Heterogeneity: I^2 = 96.4%, τ^2 = 0.8354, p < 0.0001
20 30 40 50 60 70 80 90
Prevalence of overall breastfeeding from 3 to 6 months
