# Supplementary material for: Prevalence of Breastfeeding in Infants With Down Syndrome: A Systematic Review and Meta‐Analysis
Source: Acta Paediatr. 2026 Jul 3;115(10):2063–77. doi: 10.1111/apa.70628 (PMC13552598; doi:10.1111/apa.70628)
Supplement: Supplementary file 2 [file APA-115-2063-s002.zip › apa70628-sup-0007-FigureS6.pdf]

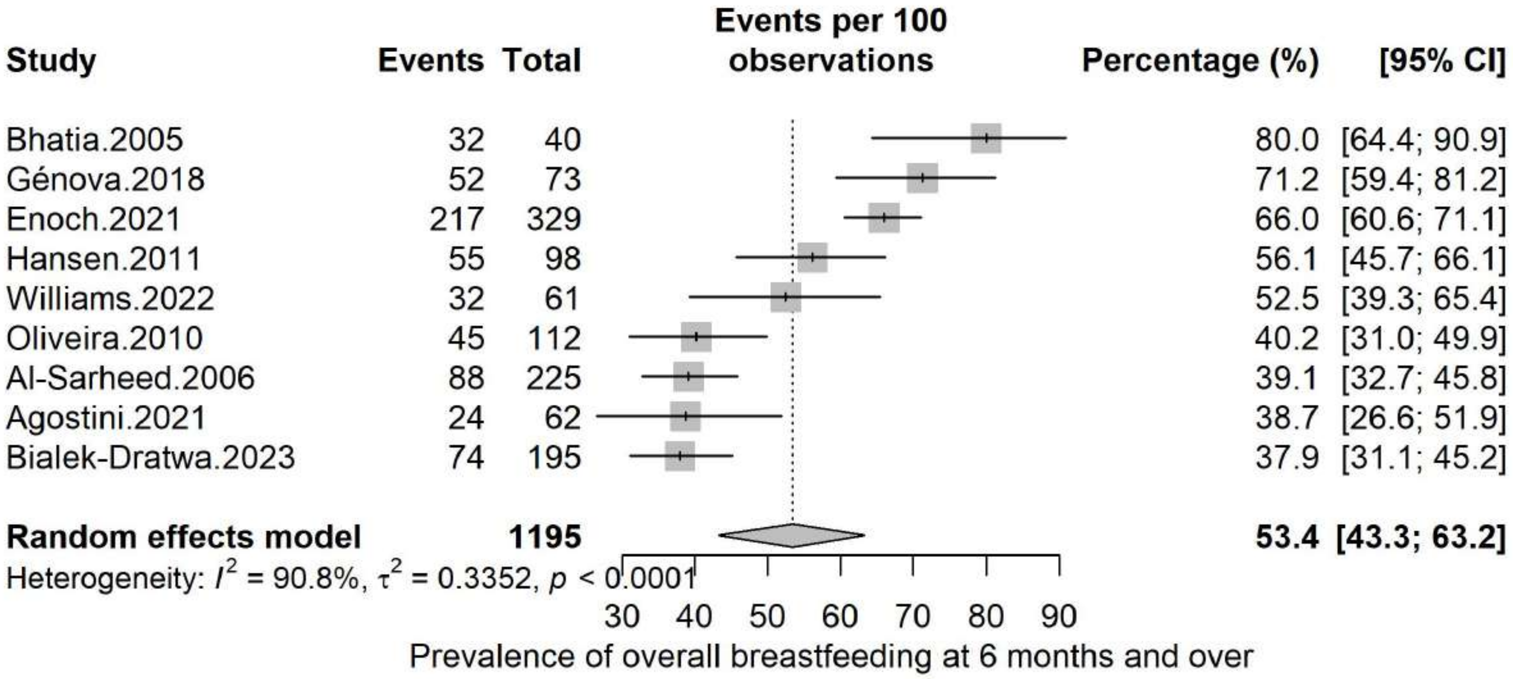
Study | Events | Total | Events per 100 observations | Percentage (%) | [95% CI]
Bhatia.2005 32 40 80.0 [64.4; 90.9]
Génova.2018 52 73 71.2 [59.4; 81.2]
Enoch.2021 217 329 66.0 [60.6; 71.1]
Hansen.2011 55 98 56.1 [45.7; 66.1]
Williams.2022 32 61 52.5 [39.3; 65.4]
Oliveira.2010 45 112 40.2 [31.0; 49.9]
Al-Sarheed.2006 88 225 39.1 [32.7; 45.8]
Agostini.2021 24 62 38.7 [26.6; 51.9]
Bialek-Dratwa.2023 74 195 37.9 [31.1; 45.2]
Random effects model 1195 53.4 [43.3; 63.2]
Heterogeneity: $I^2 = 90.8\%$, $\tau^2 = 0.3352$, $p < 0.0001$
30 40 50 60 70 80 90
Prevalence of overall breastfeeding at 6 months and over
