# Supplementary material for: Prevalence of Breastfeeding in Infants With Down Syndrome: A Systematic Review and Meta‐Analysis
Source: Acta Paediatr. 2026 Jul 3;115(10):2063–77. doi: 10.1111/apa.70628 (PMC13552598; doi:10.1111/apa.70628)
Supplement: Supplementary file 2 [file APA-115-2063-s002.zip › apa70628-sup-0010-FigureS9.pdf]

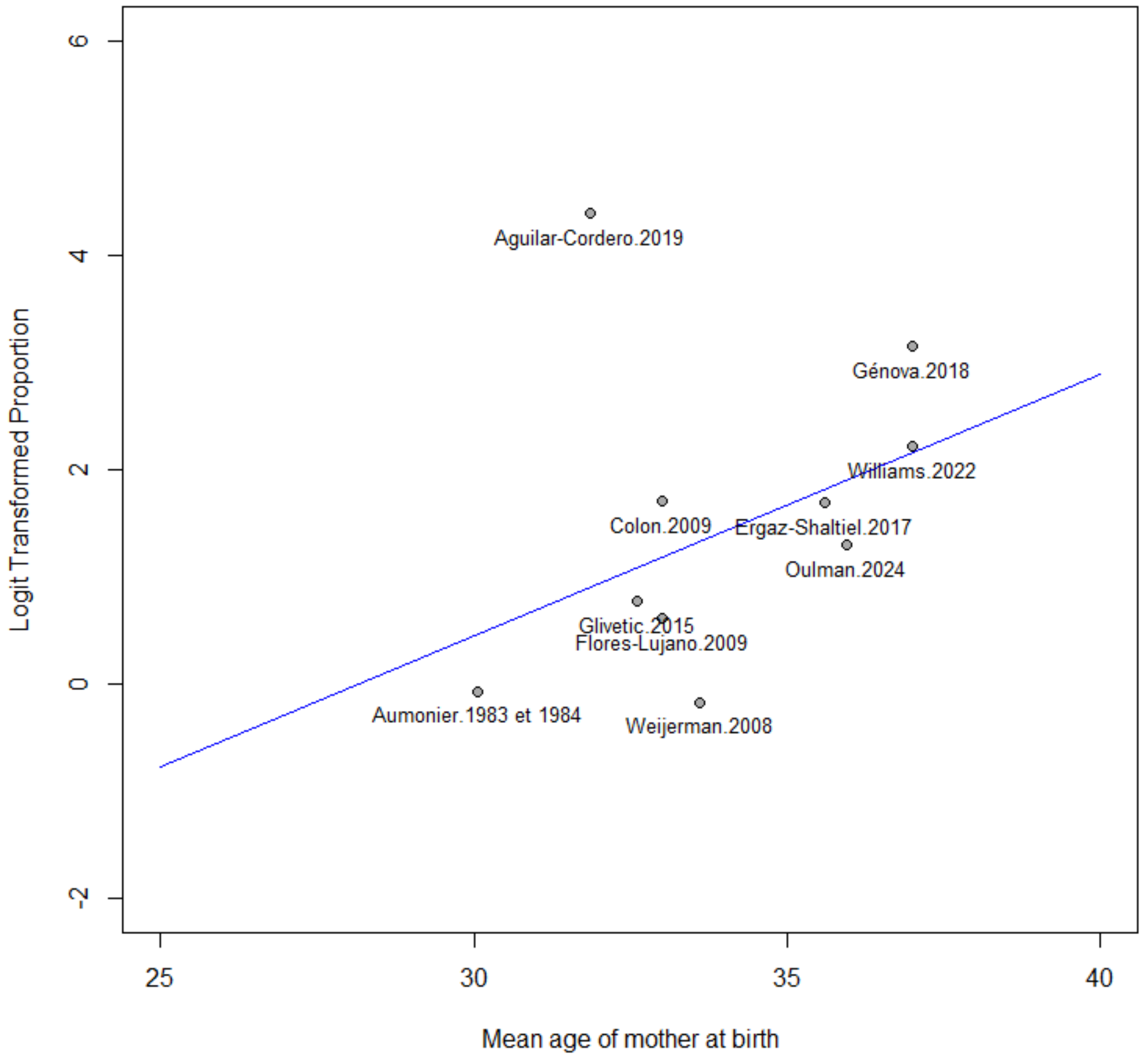
Logit Transformed Proportion
Mean age of mother at birth
-2
0
2
4
6
25
30
35
40
Aguilar-Cordero.2019
Génova.2018
Williams.2022
Colon.2009
Ergaz-Shaltiel.2017
Oulman.2024
Glivetic.2015
Flores-Lujano.2009
Aumonier.1983 et 1984
Weijerman.2008
