# Supplementary material for: Prevalence of Breastfeeding in Infants With Down Syndrome: A Systematic Review and Meta‐Analysis
Source: Acta Paediatr. 2026 Jul 3;115(10):2063–77. doi: 10.1111/apa.70628 (PMC13552598; doi:10.1111/apa.70628)
Supplement: Supplementary file 2 [file APA-115-2063-s002.zip › apa70628-sup-0011-FigureS10.pdf]

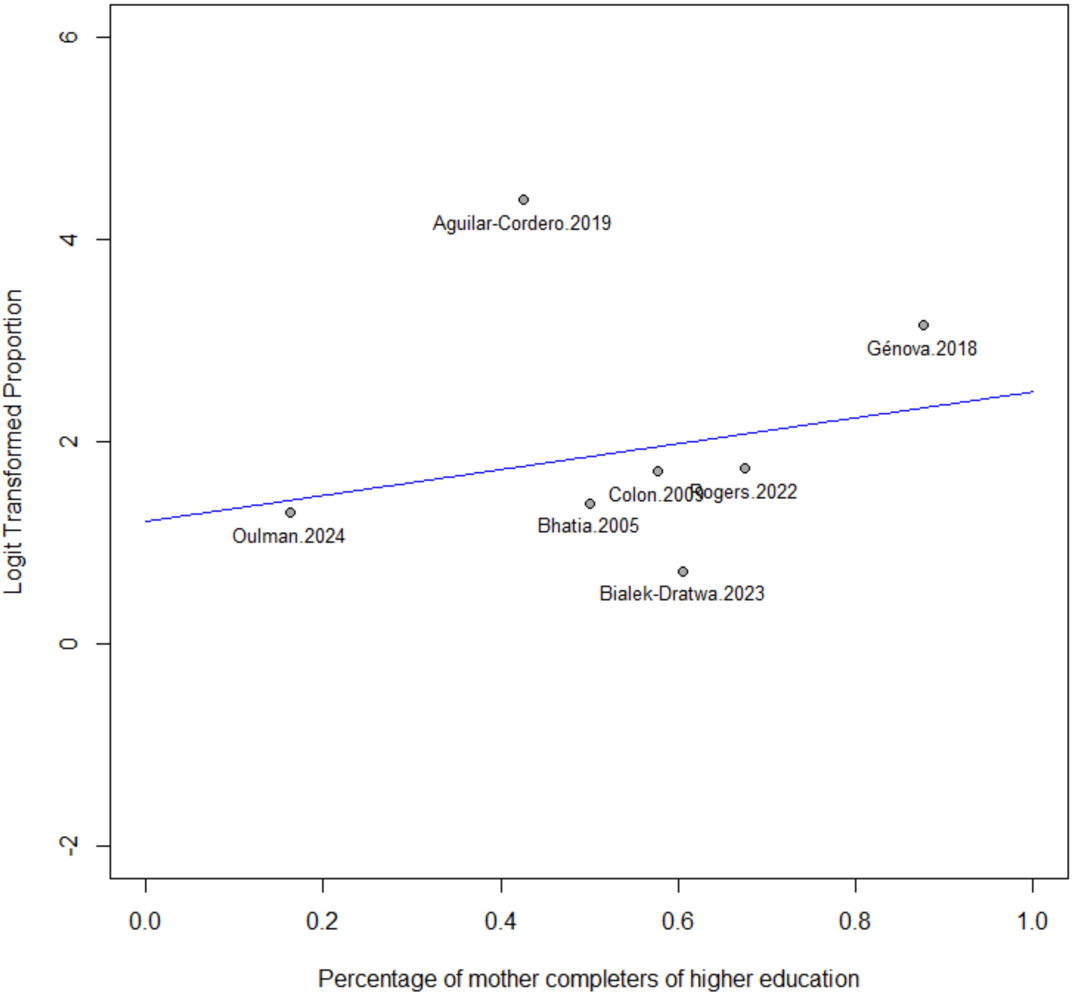

Aguilar-Cordero.2019
Génova.2018
Colon.2009
Rogers.2022
Bhatia.2005
Oulman.2024
Bialek-Dratwa.2023
-2
0
2
4
6
0.0
0.2
0.4
0.6
0.8
1.0
Logit Transformed Proportion
Percentage of mother completers of higher education
