# Supplementary material for: Prevalence of Breastfeeding in Infants With Down Syndrome: A Systematic Review and Meta‐Analysis
Source: Acta Paediatr. 2026 Jul 3;115(10):2063–77. doi: 10.1111/apa.70628 (PMC13552598; doi:10.1111/apa.70628)
Supplement: Supplementary file 2 [file APA-115-2063-s002.zip › apa70628-sup-0013-FigureS12.pdf]

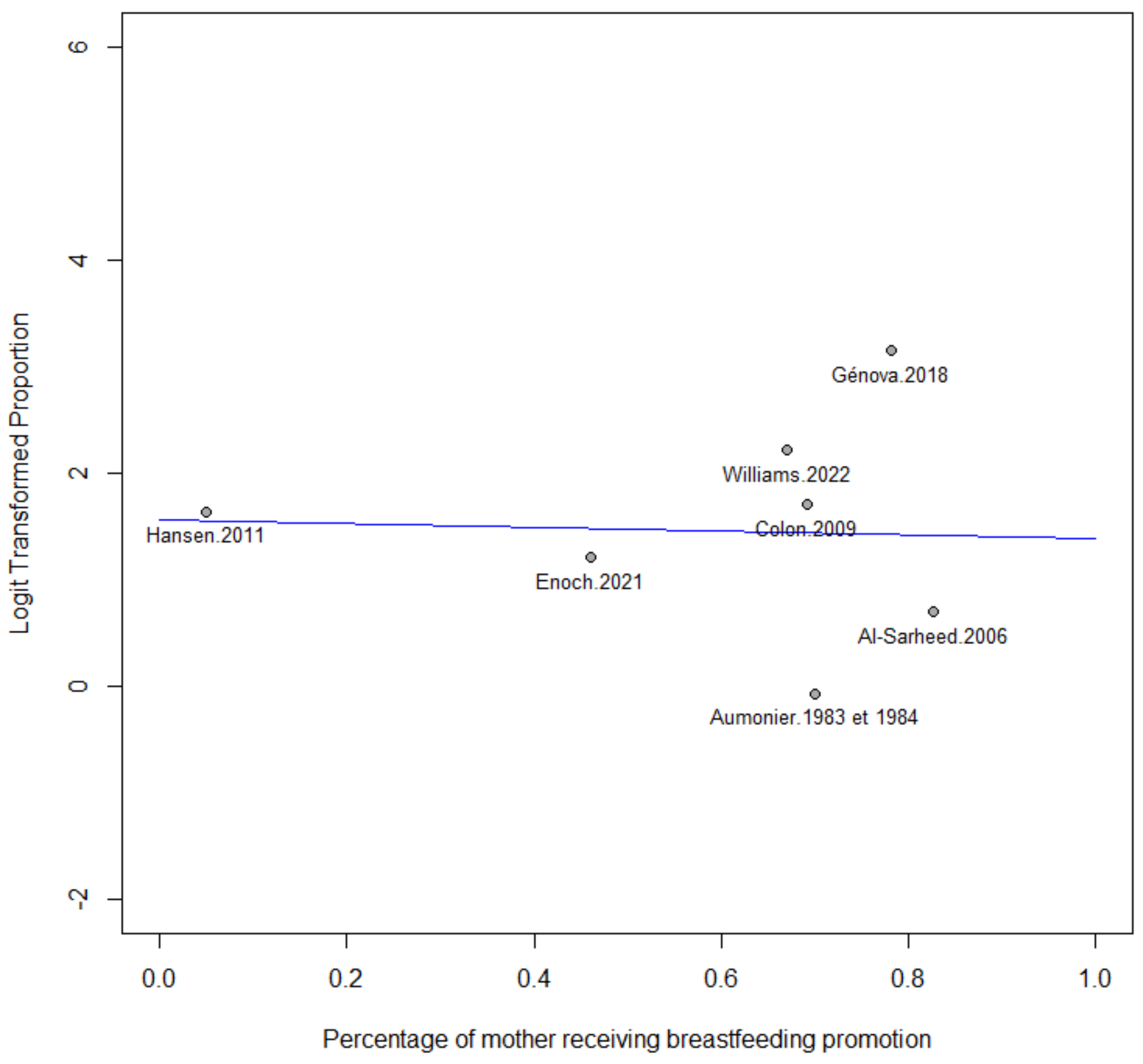
Logit Transformed Proportion
6
4
2
0
-2
Génova.2018
Williams.2022
Colon.2009
Hansen.2011
Enoch.2021
Al-Sarheed.2006
Aumonier.1983 et 1984
0.0
0.2
0.4
0.6
0.8
1.0
Percentage of mother receiving breastfeeding promotion
