# Supplementary material for: Prevalence of Breastfeeding in Infants With Down Syndrome: A Systematic Review and Meta‐Analysis
Source: Acta Paediatr. 2026 Jul 3;115(10):2063–77. doi: 10.1111/apa.70628 (PMC13552598; doi:10.1111/apa.70628)
Supplement: Supplementary file 2 [file APA-115-2063-s002.zip › apa70628-sup-0014-FigureS13.pdf]

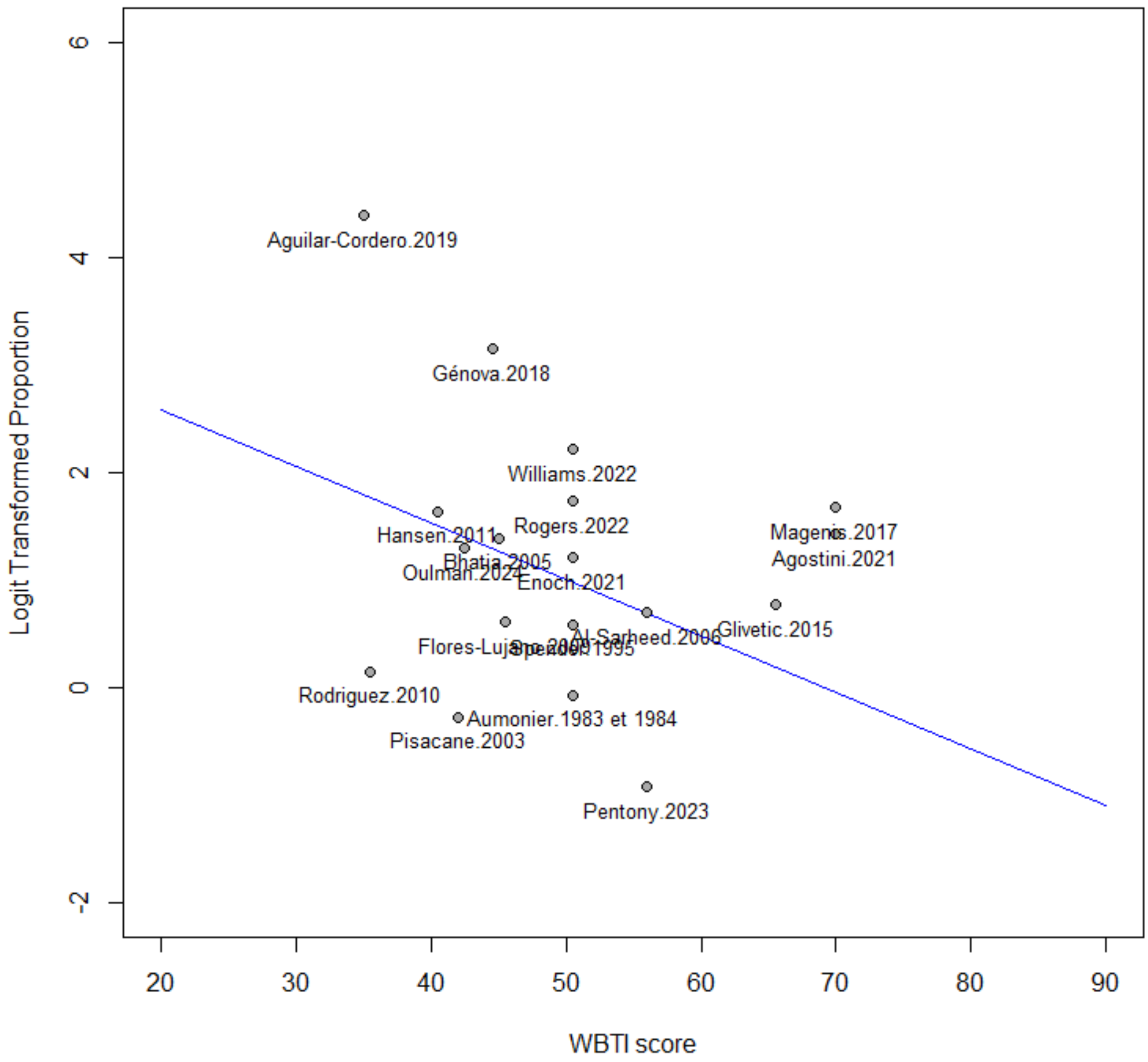

Logit Transformed Proportion
WBTI score
-2
0
2
4
6
20
30
40
50
60
70
80
90
Aguilar-Cordero.2019
Génova.2018
Williams.2022
Rogers.2022
Hansen.2011
Bhatia.2005
Oulman.2024
Enoch.2021
Agostini.2021
Glivetic.2015
Rodriguez.2010
Aumonier.1983 et 1984
Pisacane.2003
Pentony.2023
