# Supplementary material for: Prevalence of Breastfeeding in Infants With Down Syndrome: A Systematic Review and Meta‐Analysis
Source: Acta Paediatr. 2026 Jul 3;115(10):2063–77. doi: 10.1111/apa.70628 (PMC13552598; doi:10.1111/apa.70628)
Supplement: Supplementary file 2 [file APA-115-2063-s002.zip › apa70628-sup-0015-FigureS14.pdf]

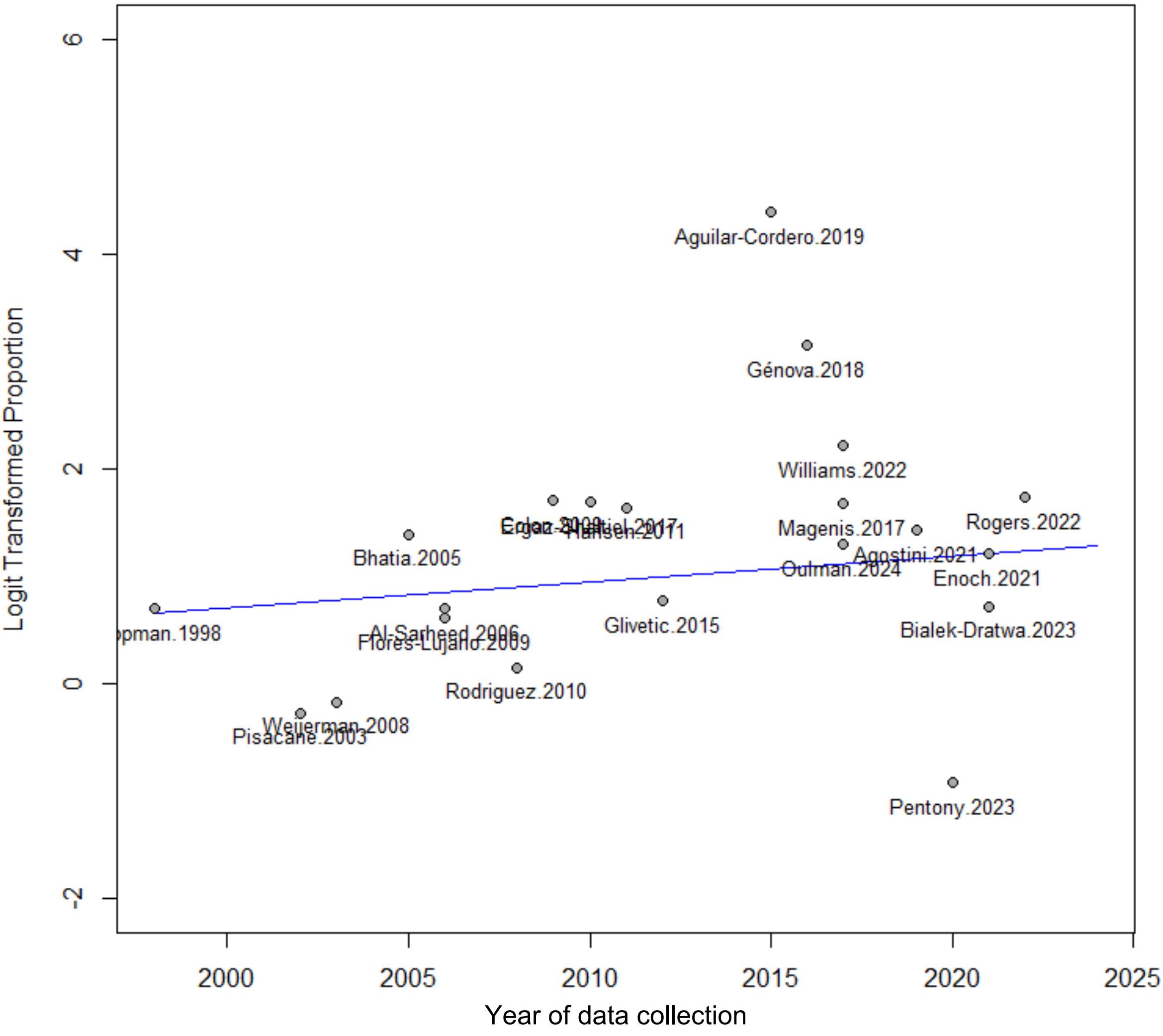

Aguilar-Cordero.2019
Génova.2018
Williams.2022
Magenis.2017
Rogers.2022
Hansen.2011
Bhatia.2005
Agostini.2021
Oulman.2024
Enoch.2021
Glivetic.2015
Bialek-Dratwa.2023
Al-Sarheed.2006
Flores-Lujano.2009
Rodriguez.2010
Weijerman.2008
Pisacane.2003
Pentony.2023
6
4
2
0
-2
2000
2005
2010
2015
2020
2025
Logit Transformed Proportion
Year of data collection
