# Supplementary material for: Prevalence of Breastfeeding in Infants With Down Syndrome: A Systematic Review and Meta‐Analysis
Source: Acta Paediatr. 2026 Jul 3;115(10):2063–77. doi: 10.1111/apa.70628 (PMC13552598; doi:10.1111/apa.70628)
Supplement: Supplementary file 2 [file APA-115-2063-s002.zip › apa70628-sup-0017-FigureS16.pdf]

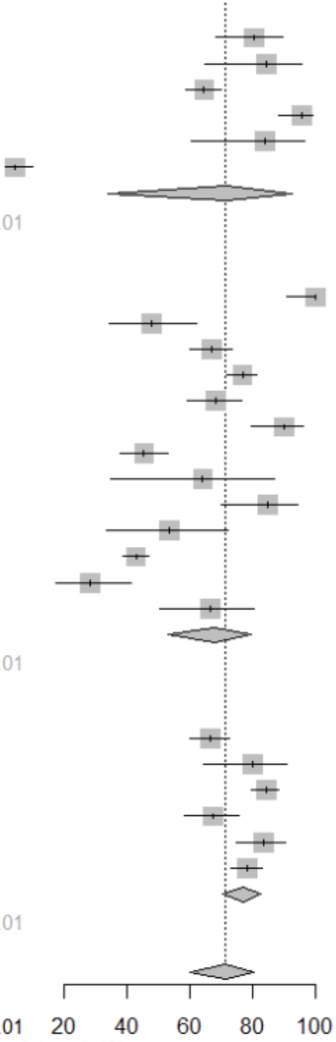

Study	Events	Total	Prop. (%)	[95% CI]
**south am.**				
Agostini.2021	50	62	80.6	[68.6; 89.6]
Colon.2009	22	26	84.6	[65.1; 95.6]
Flores-Lujano.2009	178	275	64.7	[58.8; 70.4]
Génova.2018	70	73	95.9	[88.5; 99.1]
Magenis.2017	16	19	84.2	[60.4; 96.6]
Da Cruz.2024	5	112	4.5	[1.5; 10.1]
**Random effects model**		679	72.0	[34.2; 92.7]
Heterogeneity: $I^2 = 95\%$, $\tau^2 = 3.7616$, $p < 0.01$				
**europe**				
Aguilar-Cordero.2019	40	40	100.0	[91.2; 100.0]
Aumonier.1983 et 1984	26	54	48.1	[34.3; 62.2]
Bialek-Dratwa.2023	131	195	67.2	[60.1; 73.7]
Enoch.2021	253	329	76.9	[72.0; 81.3]
Glivetic.2015	82	120	68.3	[59.2; 76.5]
Williams.2022	55	61	90.2	[79.8; 96.3]
Weijerman.2008	83	182	45.6	[38.2; 53.1]
Spender.1995	9	14	64.3	[35.1; 87.2]
Rogers.2022	34	40	85.0	[70.2; 94.3]
Rodriguez.2010	15	28	53.6	[33.9; 72.5]
Pisacane.2003	241	560	43.0	[38.9; 47.3]
Pentony.2023	17	60	28.3	[17.5; 41.4]
Hoppman.1998	28	42	66.7	[50.5; 80.4]
**Random effects model**		1725	68.0	[53.4; 79.7]
Heterogeneity: $I^2 = 93\%$, $\tau^2 = 1.1477$, $p < 0.01$				
**other**				
Al-Sarheed.2006	150	225	66.7	[60.1; 72.8]
Bhatia.2005	32	40	80.0	[64.4; 90.9]
Ergaz-Shaltiel.2017	254	301	84.4	[79.8; 88.3]
Murdoch.1991	79	117	67.5	[58.2; 75.9]
Hansen.2011	82	98	83.7	[74.8; 90.4]
Oulman.2024	218	278	78.4	[73.1; 83.1]
**Random effects model**		1059	77.2	[70.7; 82.6]
Heterogeneity: $I^2 = 84\%$, $\tau^2 = 0.1329$, $p < 0.01$				
**Random effects model**		**3351**	**71.6**	**[60.3; 80.7]**

Events per 100 observations

Heterogeneity: $I^2 = 94\%$, $\tau^2 = 1.5333$, $p < 0.01$
Test for subgroup differences: $\chi^2_2 = 1.74$, df = 2 ($p = 0.42$)
