# Supplementary material for: Prevalence of Breastfeeding in Infants With Down Syndrome: A Systematic Review and Meta‐Analysis
Source: Acta Paediatr. 2026 Jul 3;115(10):2063–77. doi: 10.1111/apa.70628 (PMC13552598; doi:10.1111/apa.70628)
Supplement: Supplementary file 2 [file APA-115-2063-s002.zip › apa70628-sup-0018-FigureS17.pdf]

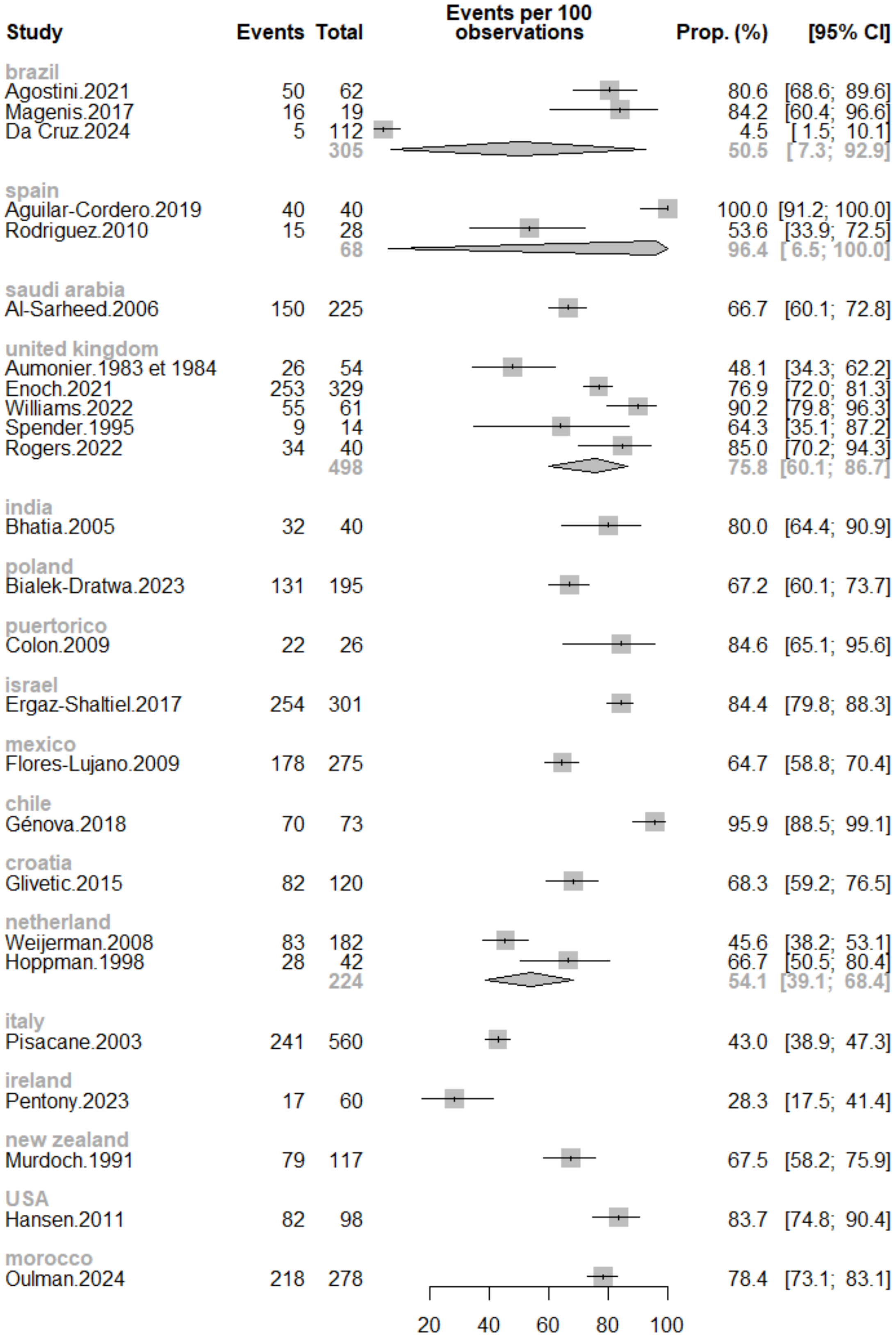
Study
Events
Total
Events per 100 observations
Prop. (%)
[95% CI]
brazil
Agostini.2021 50 62 80.6 [68.6; 89.6]
Magenis.2017 16 19 84.2 [60.4; 96.6]
Da Cruz.2024 5 112 4.5 [1.5; 10.1]
305 50.5 [7.3; 92.9]
spain
Aguilar-Cordero.2019 40 40 100.0 [91.2; 100.0]
Rodriguez.2010 15 28 53.6 [33.9; 72.5]
68 96.4 [6.5; 100.0]
saudi arabia
Al-Sarheed.2006 150 225 66.7 [60.1; 72.8]
united kingdom
Aumonier.1983 et 1984 26 54 48.1 [34.3; 62.2]
Enoch.2021 253 329 76.9 [72.0; 81.3]
Williams.2022 55 61 90.2 [79.8; 96.3]
Spender.1995 9 14 64.3 [35.1; 87.2]
Rogers.2022 34 40 85.0 [70.2; 94.3]
498 75.8 [60.1; 86.7]
india
Bhatia.2005 32 40 80.0 [64.4; 90.9]
poland
Bialek-Dratwa.2023 131 195 67.2 [60.1; 73.7]
puertorico
Colon.2009 22 26 84.6 [65.1; 95.6]
israel
Ergaz-Shaltiel.2017 254 301 84.4 [79.8; 88.3]
mexico
Flores-Lujano.2009 178 275 64.7 [58.8; 70.4]
chile
Génova.2018 70 73 95.9 [88.5; 99.1]
croatia
Glivetic.2015 82 120 68.3 [59.2; 76.5]
netherland
Weijerman.2008 83 182 45.6 [38.2; 53.1]
Hoppman.1998 28 42 66.7 [50.5; 80.4]
224 54.1 [39.1; 68.4]
italy
Pisacane.2003 241 560 43.0 [38.9; 47.3]
ireland
Pentony.2023 17 60 28.3 [17.5; 41.4]
new zealand
Murdoch.1991 79 117 67.5 [58.2; 75.9]
USA
Hansen.2011 82 98 83.7 [74.8; 90.4]
morocco
Oulman.2024 218 278 78.4 [73.1; 83.1]
20
40
60
80
100
