# Supplementary material for: Prevalence of Breastfeeding in Infants With Down Syndrome: A Systematic Review and Meta‐Analysis
Source: Acta Paediatr. 2026 Jul 3;115(10):2063–77. doi: 10.1111/apa.70628 (PMC13552598; doi:10.1111/apa.70628)
Supplement: Supplementary file 2 [file APA-115-2063-s002.zip › apa70628-sup-0019-FigureS18.pdf]

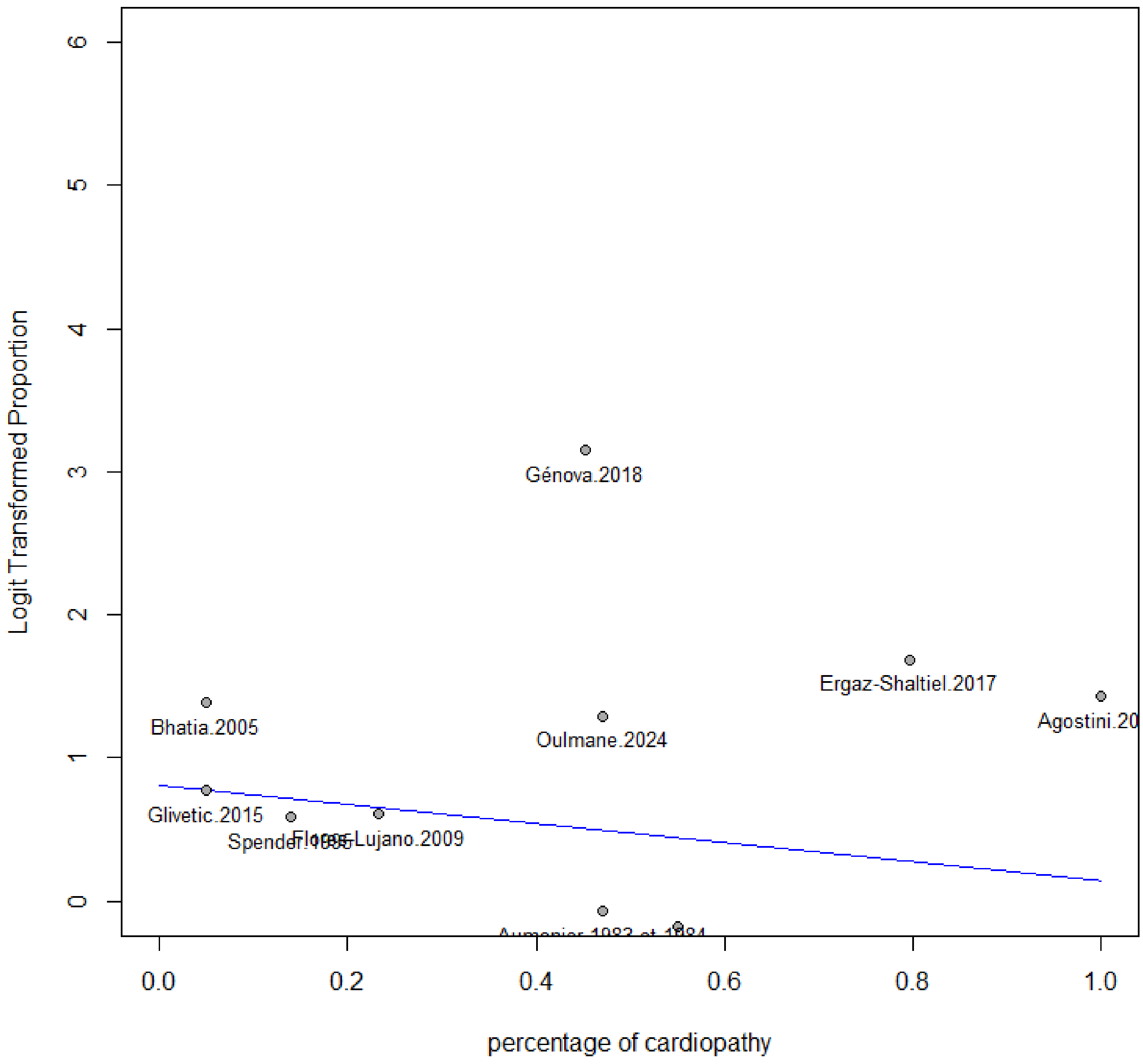
Logit Transformed Proportion
0
1
2
3
4
5
6
Génova.2018
Ergaz-Shaltiel.2017
Bhatia.2005
Oulmane.2024
Agostini.20
Glivetic.2015
Spender
Flores-Lujano.2009
0.0
0.2
0.4
0.6
0.8
1.0
percentage of cardiopathy
